# Human Cancer Cells Signal Their Competitive Fitness Through MYC Activity

**DOI:** 10.1038/s41598-017-13002-1

**Published:** 2017-10-03

**Authors:** Simone Di Giacomo, Manuela Sollazzo, Dario de Biase, Moira Ragazzi, Paola Bellosta, Annalisa Pession, Daniela Grifoni

**Affiliations:** 1 Department of Pharmacy and Biotechnology, University of Bologna, Via Selmi 3, Bologna, 40126 Italy; 2 Pathology Unit, IRCCS Arcispedale Santa Maria Nuova, Via Amendola 2, 42122 Reggio Emilia, Italy; 3 Center for Integrate Biology (CIBIO), University of Trento, Via Sommarive 9, Povo, (TN) 38123 Italy

## Abstract

MYC-mediated cell competition is a cell-cell interaction mechanism known to play an evolutionary role during development from *Drosophila* to mammals. Cells expressing low levels of MYC, called *losers*, are committed to die by nearby cells with high MYC activity, called *winners*, that overproliferate to compensate for cell loss, so that the fittest cells be selected for organ formation. Given MYC’s consolidated role in oncogenesis, cell competition is supposed to be relevant to cancer, but its significance in human malignant contexts is largely uncharacterised. Here we show stereotypical patterns of MYC-mediated cell competition in human cancers: MYC-upregulating cells and apoptotic cells were indeed repeatedly found at the tumour-stroma interface and within the tumour parenchyma. Cell death amount in the stromal compartment and MYC protein level in the tumour were highly correlated regardless of tumour type and stage. Moreover, we show that MYC modulation in heterotypic co-cultures of human cancer cells is sufficient as to subvert their competitive state, regardless of genetic heterogeneity. Altogether, our findings suggest that the innate role of MYC-mediated cell competition in development is conserved in human cancer, with malignant cells using MYC activity to colonise the organ at the expense of less performant neighbours.

## Introduction

MYC family proteins are subject to continuous investigation due to their implication in most, if not all, cellular vital processes. They are found deregulated in pathological conditions such as cancer, where they play essential roles in reprogramming gene expression, ultimately promoting metabolic shift, cell growth and proliferation^1^. MYC proteins have also proven to trigger non cell-autonomous responses at the tissue/organ level^2,3^ which deeply impact cancer outcome, according to the emerging vision of cancer as a social network of interplaying cells^4^. Among these non cell-autonomous mechanisms, MYC-Mediated Cell Competition (MMCC) has been shown to play an essential role in development, conserved from *Drosophila* to mammals, carried out through a meticulous selection of the cells contributing to the final organs^5–8^. This process is based on the mutual comparison of MYC activity by adjacent cells: as soon as cells showing low levels of MYC activity (called *losers*) are detected in a field inhabited by cells with high MYC expression (called *winners*), molecular interactions are elicited that finally lead to the apoptotic elimination of the weaker cells. As a consequence, cells with high MYC protein levels overproliferate to compensate for tissue loss^9^. Conservation of this complex phenomenon across distant species is not surprising, as *Drosophila* and mammalian MYC are known to substitute each other functions in several contexts^10,11^. MMCC has also been demonstrated to be active in a number of different organs and tissues, from the above cited epithelium to brain cells^12^, cardiomyocytes^13,14^ and fibroblasts^15^, therefore it seems to work regardless of cell histotype. Moreover, MYC and cell competition have been found involved in several models of cancer growth in *Drosophila*, from the most studied wing disc^16–19^ to the recently explored intestinal cancer^20^, and in human cancer cell lines^21^. c-MYC is frequently up-regulated in epithelial tumours^22^, paving the way to elegant speculations about a relevance for MMCC to human cancer biology^23,24^. In *Drosophila*, MYC up-regulation is induced, among others, by the downstream effector of the Hippo pathway Yorkie (Yki)^25,26^, the fly homologue of the human Yes-Associated Protein (YAP), found aberrantly activated in many human cancers^27^. Disruption of cell polarity proteins is associated with Yki/YAP activation^28,29^; deep alterations of the Lgl (Lethal giant larvae) polarity protein have been found to promote Yki nuclear translocation, MYC up-regulation and tumourigenesis in epithelial tissues^16,17,30–32^. We previously showed that the human homologue of *Drosophila lgl*, namely *HUGL-1(* *HU* *uman* *G* *iant* *L* *arvae)*, is functionally conserved^33^, and after that study a numer of groups associated its reduced expression or protein delocalisation with cancer progression in many different organs^33–41^. Therefore, based on this evidence collected both in *Drosophila* and mammals, we first explored the presence and function of MMCC in human cancer tissues. According to its evolutionary conservation in development, we found stereotypical patterns of MMCC in a variety of human cancer samples, from *in situ* lesions to metastases, occurring both at the tumour/stroma interface and within the tumour parenchyma. As human cancers can display startling genetic diversification, we then investigated a possible role of MMCC in clone selection by carrying out competition assays in heterotypic co-cultures of human cancer cell lines. We found that, whatever the genetic background of the co-plated cells, modulation of MYC activity was sufficient as to subvert their competitive behaviour. Our findings suggest that MMCC may be an innate mechanism, conserved from developmento to cancer, contributing to cell selection and expansion during growth.

## Results

### Human cancers display stereotypical patterns of MYC-mediated cell competition

**Figure 1:**
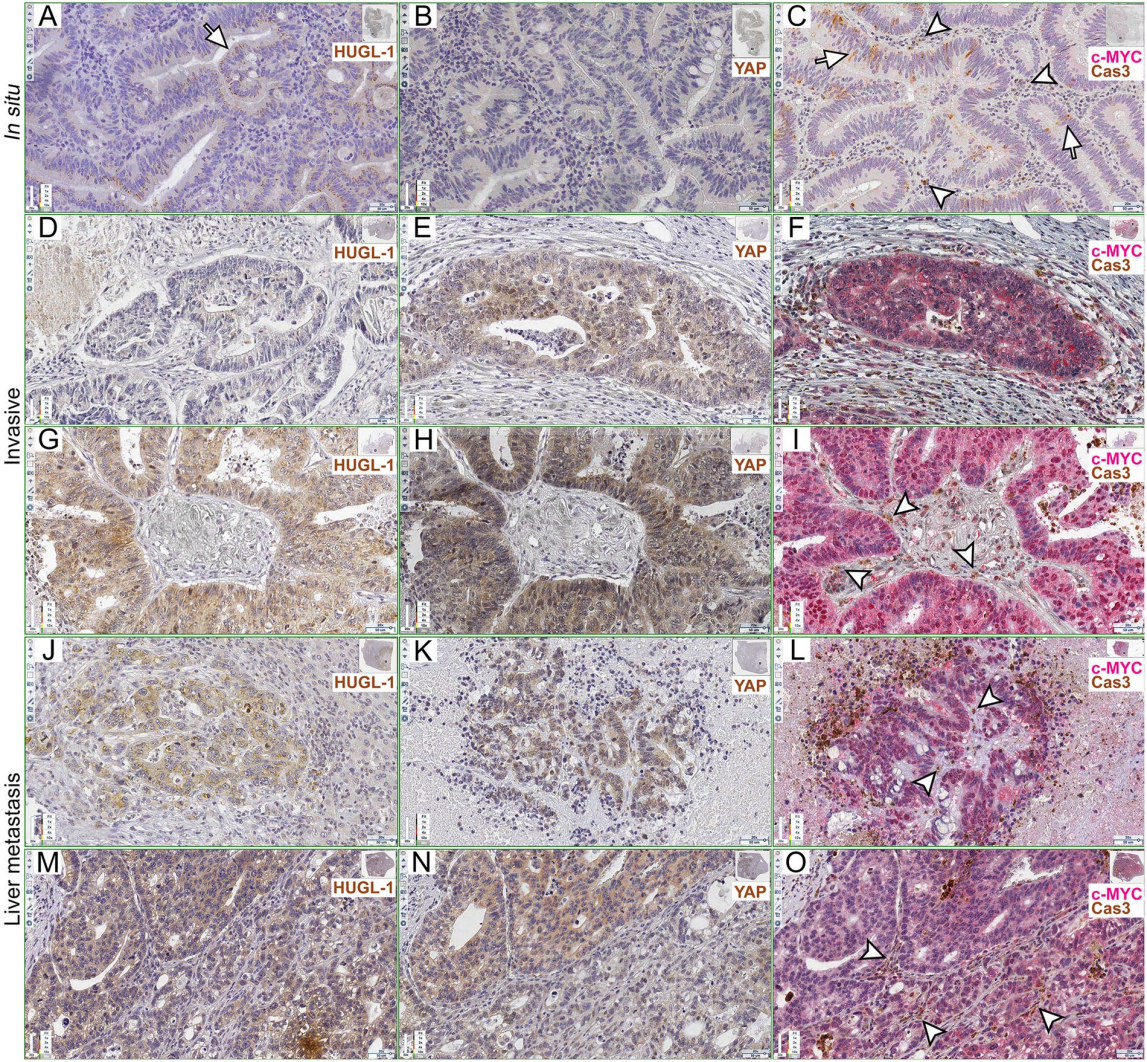
Human colon cancers display apoptotic cell death at the tumour/stroma interface. (**A**–**C**) HUGL-1 (**A**), YAP (**B**) and c-MYC/Cas3 (**C**) staining of an *in situ* carcinoma. The arrow in (**A**) highlights a region of residual HUGL-1 protein localisation. In (**C**), the arrows indicate the presence of Cas3-positive cells within the transforming epithelium, while the arrowheads point to apoptotic stromal cells. (**D**–**I**) HUGL-1 (**D**,**G**), YAP (**E**,**H**) and c-MYC/Cas3 (**F**,**I**) staining of invasive carcinomas. The arrowheads in *I* indicate some stromal cells undergoing apoptotic death. (**J**–**O**) HUGL-1 (**J**,**M**), YAP (**K**,**N**) and c-MYC/Cas3 (**L**,**O**) staining of metastatic cancers. The arrowheads in (**L** and **O**) indicate Cas-3 positive stromal cells. Scale bars are indicated in each frame.

**Figure 2:**
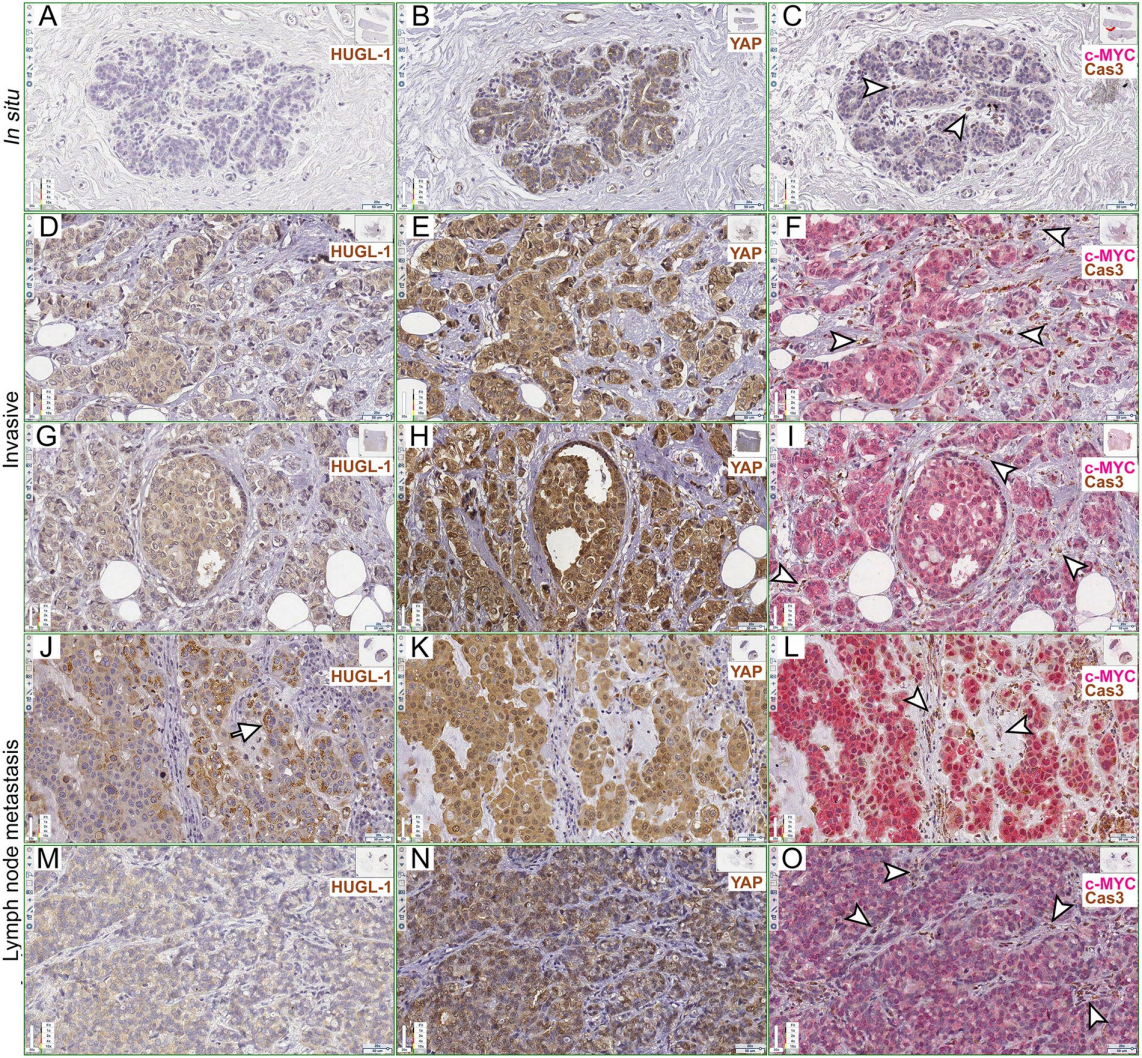
Apoptotic cell death at the tumour/stroma interface in human breast cancers. (**A**–**C**) HUGL-1 (**A**), YAP (**B**) and c-MYC/Cas3 (**C**) staining of an *in situ* carcinoma. The arrowheads in (**C**) highlight some stromal cells positive to Cas3 staining. (**D**–**I**) HUGL-1 (**D**,**G**), YAP (**E**,**H**) and c-MYC/Cas3 (**F**,**I**) staining of invasive carcinomas. The arrowheads in (**F** and **I**) indicate some stromal cells undergoing apoptotic death. (**J**–**O**) HUGL-1 (**J**,**M**), YAP (**K**,**N**) and c-MYC/Cas3 (**L**,**O**) staining of metastatic cancers. The arrow in (**J**) highlights a region of residual HUGL-1 protein localisation. The arrowheads in (**L** and **O**) indicate Cas-3 positive stromal cells. Scale bars are indicated in each frame.

**Figure 3:**
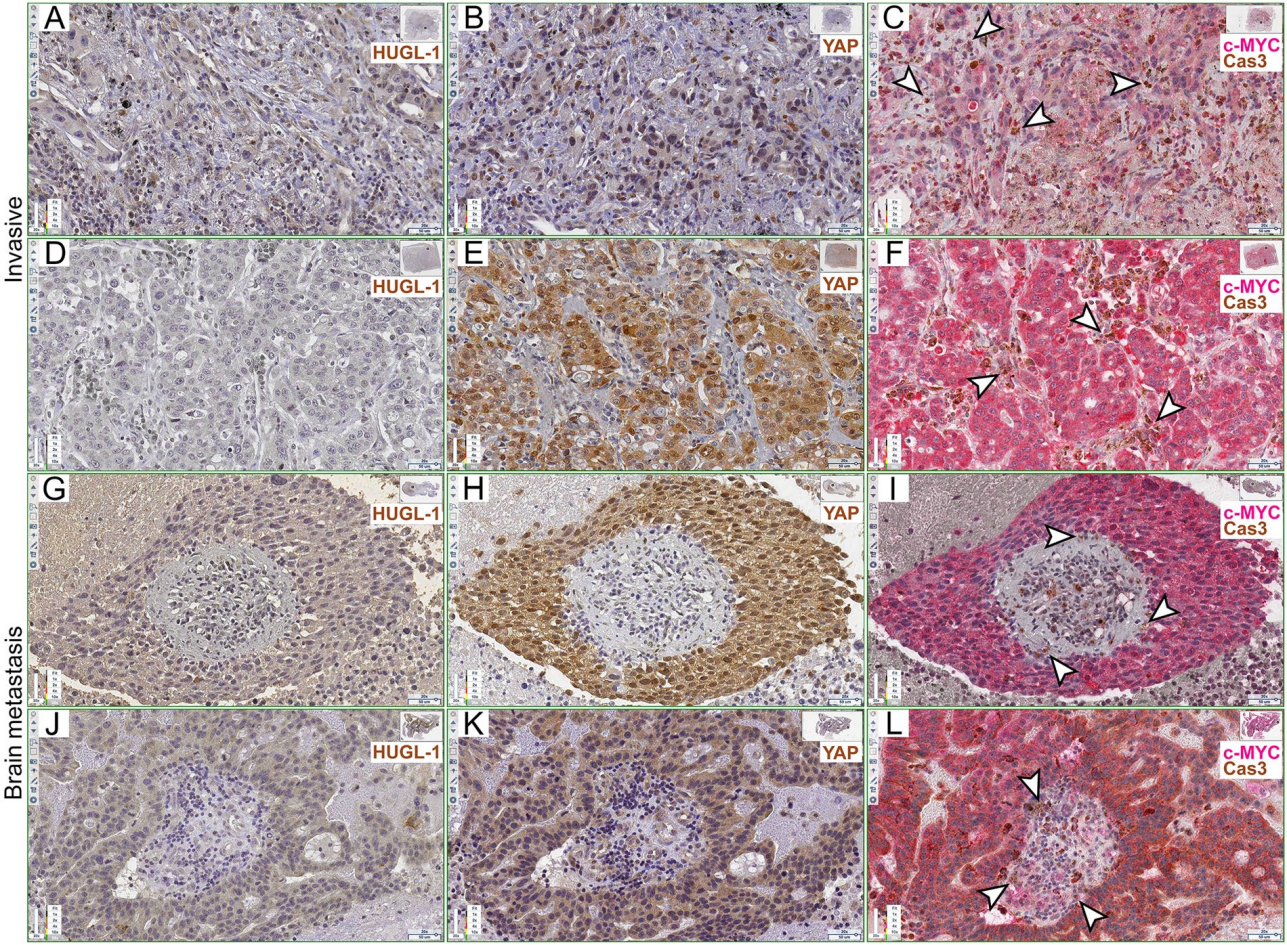
Human lung cancers show Caspase 3-positive stromal cells at the tumour border. (**A**–**F**) HUGL-1 (**A**,**D**), YAP (**B**,**E**) and c-MYC/Cas3 (**C**,**F**) staining of invasive carcinomas. The arrowheads in (**C** and **F**) highlight some stromal cells positive to Cas3 staining. (**G**–**L**) HUGL-1 (**G**,**J**), YAP (**H**,**K**) and c-MYC/Cas3 (**I**,**L**) staining of metastatic tumours. The arrowheads in (**I** and **L**) indicate Cas-3 positive stromal cells. Scale bars are indicated in each frame.

**Figure 4:**
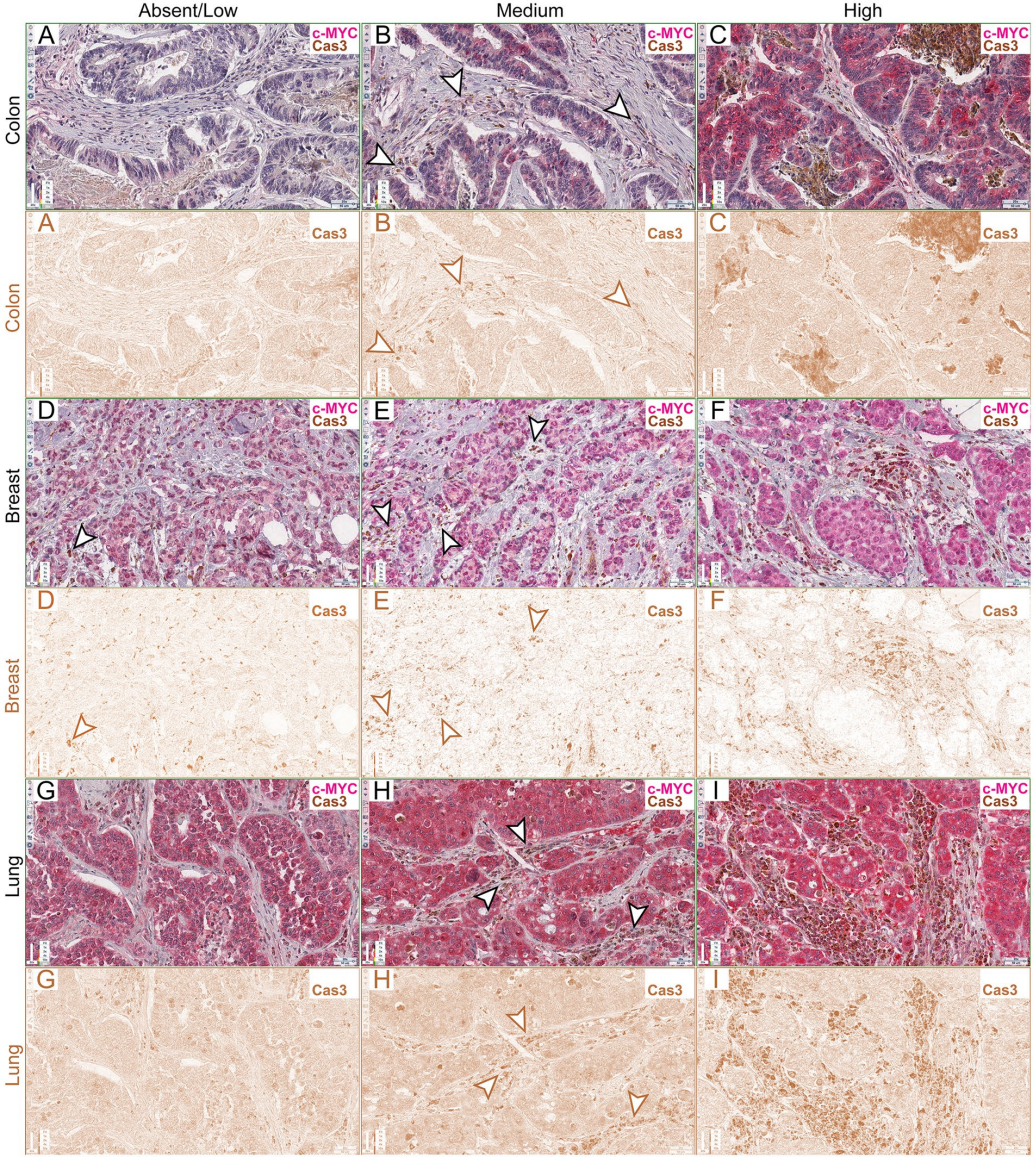
Caspase 3 quantification in human cancer samples. (**A**–**I**) c-MYC and Cas3 staining of colon (**A**–**C**), breast (**D**–**F**) and lung (**G**–**I**) human cancer samples. For each sample, the original RGB image (upper panels) and the deconvolved DAB image (lower panels) are shown. The arrowheads in (**B**,**D**,**E** and **H**) highlight stromal cells positive to Cas3 staining. In (**C**,**F** and **I**) cell death is self-evident. Scale bars are indicated in each frame.

**Figure 5:**
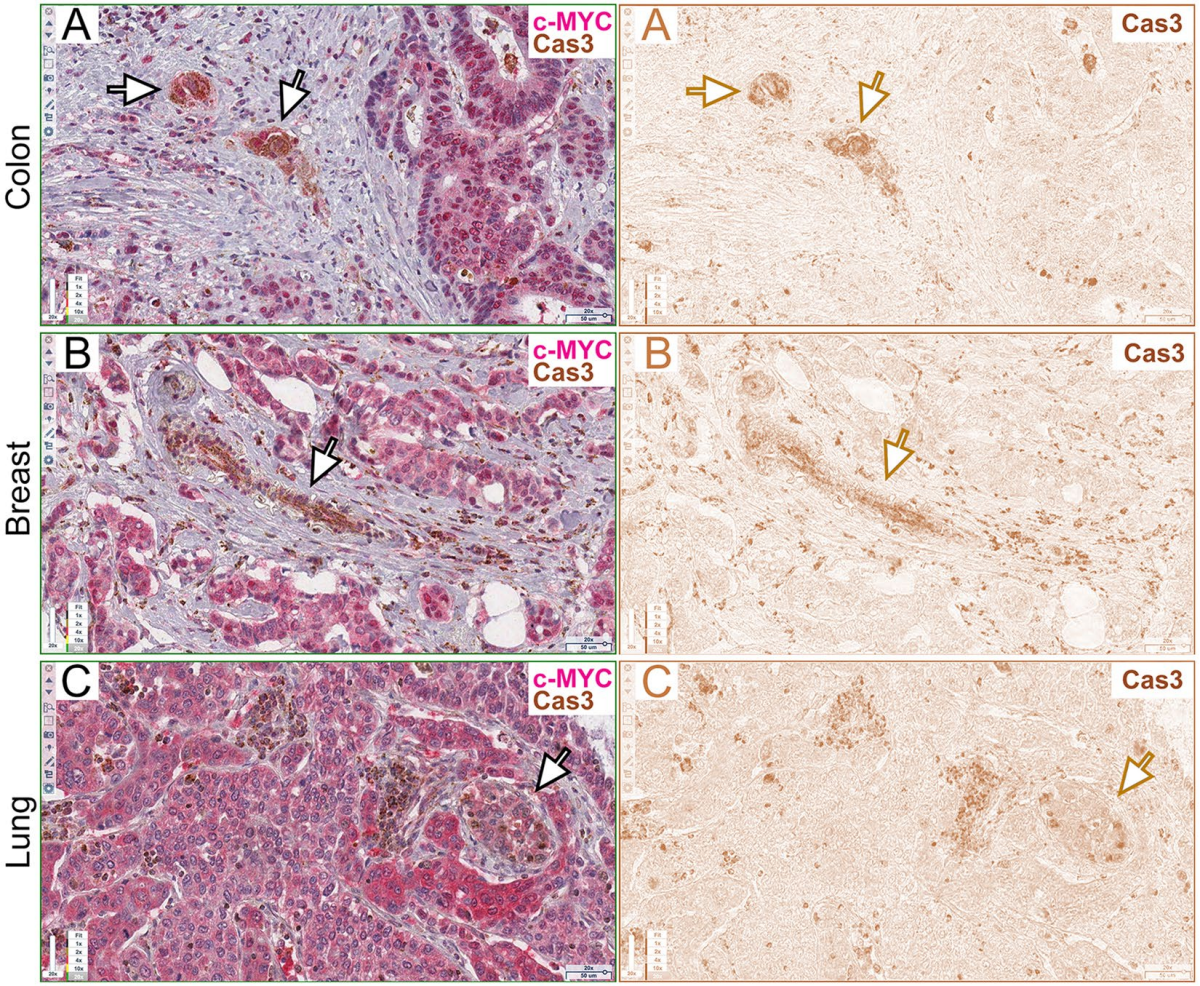
Human cancers display self-competitive behaviour. (**A**–**C**) c-MYC and Cas3 staining of colon (**A**), breast (**B**) and lung (**C**) human cancer samples. For each sample, the original RGB image (left panel) and the deconvolved DAB image (right panel) are shown. The arrows indicate groups of tumour cells positive to Caspase 3 staining. Scale bars are indicated in each frame.

A remarkable number of studies has characterised several morphological and molecular aspects of cell competition in different species, organs, cell types and physio-pathological contexts^12,42^. We therefore decided to funnel this plenty of information towards the analysis of MMCC in human cancers. We examined a total of 27 human samples of epithelial tumours from several organs (Supplementary Table S1, columns A and B). In principle, alterations of *HUGL-1*, the human orthologue of *Drosophila lgl*, should deregulate, among others, YAP, the human Yki orthologue, thereby activating transcription of its target genes, including c-MYC^43^. This, in turn, would cause caspase-dependent out-competition of nearby cells, such as it happens in *Drosophila* models of cell competition^44–46^. We first investigated HUGL-1, YAP, c-MYC and activated Caspase 3 (hereafter referred to as Cas3) distribution in colon cancers, where *HUGL-1* alterations have been associated with malignant progression^37^. A normal colon mucosa is shown in Supplementary Figure S1, where HUGL-1 appears to be localised at cell membranes, as previously reported^37^ (Supplementary Fig. S1A, see inset), while YAP (Supplementary Fig. S1B, see inset) and c-MYC/activated Caspase 3 are barely detectable (Supplementary Fig. S1C, see insets). In Supplementary Figure S1D,E, control stainings with no primary antibodies are also shown. Figure 1 and following show sequential slices of cancer samples, with the Region Of Interest (ROI) highlighted in the upper-right thumbnail; each antibody used is identified by a color-code label and magnification is indicated in the lower-right scale bar. Figure 1A–C shows an *in situ* colon carcinoma where HUGL-1 is partly dispersed throughout the cytoplasm (the arrow in Fig. 1A indicates an example of membrane retention), YAP is mildly expressed all across the cellular volume (Fig. 1B), the tumour parenchyma expresses low levels of c-MYC (Fig. 1C) and a number of epithelial (arrows indicate some -hereafter i.s.-) and stromal cells (arrowheads i.s.) are positive to the Cas3 antibody. This may be consistent with a role for cell competition in the early steps of transformation, as an intrinsic mechanism of tumour suppression^47^. To confirm specific staining of apoptotic cells by Cas3, we carried out a TUNEL assay on normal and cancer tissues, and we obtained positive signals in the same regions as those marked by the active Caspase 3 (Supplementary Fig. S2, arrows i.s.). Figure 1D–I shows the staining for the same markers in two cases of invasive colon carcinoma. HUGL-1 appears completely released from the membrane (Fig. 1D,G), YAP shows cytoplasmic and nuclear enrichment (Fig. 1E,H), c-MYC is overexpressed (Fig. 1F,I) and a number of stromal cells at the tumour-stroma interface are positive to Cas3 (Fig. 1F,I, arrowheads in I i.s.). Similar phenotypes were observed in colon-derived liver metastasis (Fig. 1J–O), where HUGL-1 is delocalised (Fig. 1J,M), YAP is abundant in the cytoplasm and stains some cell nuclei (Fig. 1K,N) and c-MYC-positive tumour cells (Fig. 1L,O) enclose Cas3-positive stromal cells (Fig. 1L,O arrowheads i.s.). We then tested if similar behaviours were associated with cancers derived from other organs. Figure 2 displays five cases of breast cancer at progressive stages of the disease. In Fig. 1A–C, an *in situ* carcinoma shows complete loss of HUGL-1 at the cell membrane (Fig. 2A), where it is known to localise in normal breast tissue^48^, YAP (Fig. 2B) and c-MYC (Fig. 2C) are mildly expressed and few Cas3-positive fibroblasts are interspersed amid the tumour cells (Fig. 2C, arrowheads i.s.). Figure 2D–I displays two cases of invasive breast cancer where HUGL-1 appears completely released in the cytoplasm (Fig. 2D,G), YAP (Fig. 2E,H) and c-MYC (Fig. 2F,I) stain the most part of cell nuclei, and Cas3 stains a number of fibroblasts and other stromal cells at the tumour boundaries (Fig. 2F,I, arrowheads i.s.). Finally, two cases of lymph node metastasis are presented in Fig. 2J–O, where HUGL-1 is partially retained at the membrane (Fig. 2J, arrow indicates an example region) or completely cytoplasmic (Fig. 2M); YAP (Fig. 2K,N) and c-MYC (Fig. 2L,O) are highly expressed in the cancer cell nuclei, and Cas3 stains dispersed stromal cells (arrowheads i.s. in Fig. L,O). We finally examined lung cancers, where reduced expression of HUGL-1 is known to contribute to disease progression^34^. Figure 3A–F shows two cases of invasive lung cancer where HUGL-1 appears completely delocalised/lost (Fig. 3A,D), YAP and c-MYC are aberrantly expressed in the nuclei (Fig. 3B,C) or in the whole cellular volume (Fig. 3E,F) and, again, a number of Cas3-positive stromal cells are dispersed amid tumour cells (arrowheads i.s. in Fig. 3C,F). In Fig. 3G–L two cases of brain metastasis are shown where HUGL-1 localisation is completely lost (Fig. 3G,J), YAP (Fig. 3H,K) and c-MYC (Fig. 3I,L) are aberrantly expressed and many stromal cells are positive to Cas3 staining (arrowheads i.s. in Fig. 3I,L). To better visualise HUGL-1 and YAP localisation, we took advantage of a deconvolution software dedicated to IHC samples (see Methods for details) and separated the Hematoxylin staining (blue, cell nuclei) from the DAB staining (brown, HUGL-1 and YAP). The results of this analysis can be found in Supplementary Figure S3 for HUGL-1 and Supplementary Figure S4 for YAP. As can be appreciated in the Figures, HUGL-1 expression was very low and in some cases comparable to the background signal (Supplementary Figure S3, right panel), while YAP was highly expressed both in the nucleus and the cytoplasm of tumour cells (Supplementary Fig. S4, right panel), confirming what we observed in the RGB images. Additional c-MYC/Cas3 stainings can be found in Supplementary Figure S5. In particular, the images report some fields akin to *Drosophila* supercompetition^16^, with c-MYC-expressing tumour parenchyma surrounded by a crowd of stromal cells positive to Cas3 staining (Supplementary Fig. S5A,B, arrowheads indicate some stromal cells), and/or cell-cell intercalation^49^, with groups of tumour cells of variable dimensions entrapping clusters of stromal cells (Supplementary Figure S5C-F, arrowheads i.s.). Altogether, these detailed, though preliminary, observations suggest a possible correlation between loss of cell polarity (HUGL-1 decrease or delocalisation), Hippo pathway deregulation (YAP overexpression and nuclear translocation) and MYC overexpression in tumour cells, combined with apoptotic death of nearby stromal cells. With the aim to find a correlation between cell death in the stromal compartment and MYC overexpression in the tumour parenchyma, we proceeded with quantification of Caspase 3 and c-MYC signals. We first deconvolved from RGB into Hematoxylin (blue, cell nuclei), Permanent Red (pink, c-MYC) and DAB (brown, Cas3) a total 270 frames stained for c-MYC and Cas3 (10 fields/tumour sample). For Cas3 analysis, they were subdivided into three classes based on our observation. These classes were defined as “Absent/Low” (Fig. 4A,D,G), “Medium” (Fig. 4B,E,H) and “High” (Fig. 4C,F,I). Arrowheads in the figure indicate some examples of Cas3-positive cells. The class “Absent/Low” was attributed score 0, the class “Medium” score 1 and the class “High” score 2. On the basis of this classification, each tumour sample was given a Cas3-score, obtained by averaging the scores attributed to the ten frames observed. These scores can be found in Supplementary Table S1, column C. To quantify c-MYC expression in tumour cells, we used the same frames as above and calculated in ImageJ the reciprocal intensity of a representative ROI in each frame (see Methods for details). An example is given in Supplementary Figure S6, where a complete deconvolution is shown. On the basis of this calculation, each tumour sample was attributed a c-MYC score, which was also in this case obtained by averaging the scores attributed to the ten frames analysed. These scores can be found in Supplementary Table S1, column D. Cas3 and c-MYC scores underwent a Pearson’s correlation coefficient calculation and, as can be seen in Supplementary Table S1, column E, R was 0.9743 for colon cancers, 0.986 for breast cancers and 0.989 for lung cancers, with p > 0.001. This means that in the tumour types analysed, Cas3 signal in the stromal compartment was highly correlated to c-MYC protein levels in the tumour parenchyma, supporting the existence of a basic signature of MMCC in human cancers. In addition to the elimination of stromal cells, which recurred in all the samples analysed, we were also able to observe Cas3-positive tumour cells close to or surrounded by high c-MYC expressing cells. In Fig. 5, examples of this behaviour are shown in colon cancer (Fig. 5A, arrows indicate Cas3-positive tumour cells), breast cancer (Fig. 5B, the arrow points to a group of dying tumour cells) and lung cancer (Fig. 5C). In this latter image, the arrow indicates a cluster of apoptotic cells showing a c-MYC level comparable to that of other tumour cells in the field; it is however “embraced” by higher c-MYC-expressing cells, and this may cause its elimination^50^. These latter observations suggest that MMCC, further to promote the expansion of c-MYC-expressing tumour cells at the expense of the surrounding stromal cells, may shape human tumour microevolution through constant culling of cells with the lowest c-MYC activity in the field.

### Modulation of c-MYC activity in heterotypic co-cultures of human cancer cells alters their competitive state

**Figure 6:**
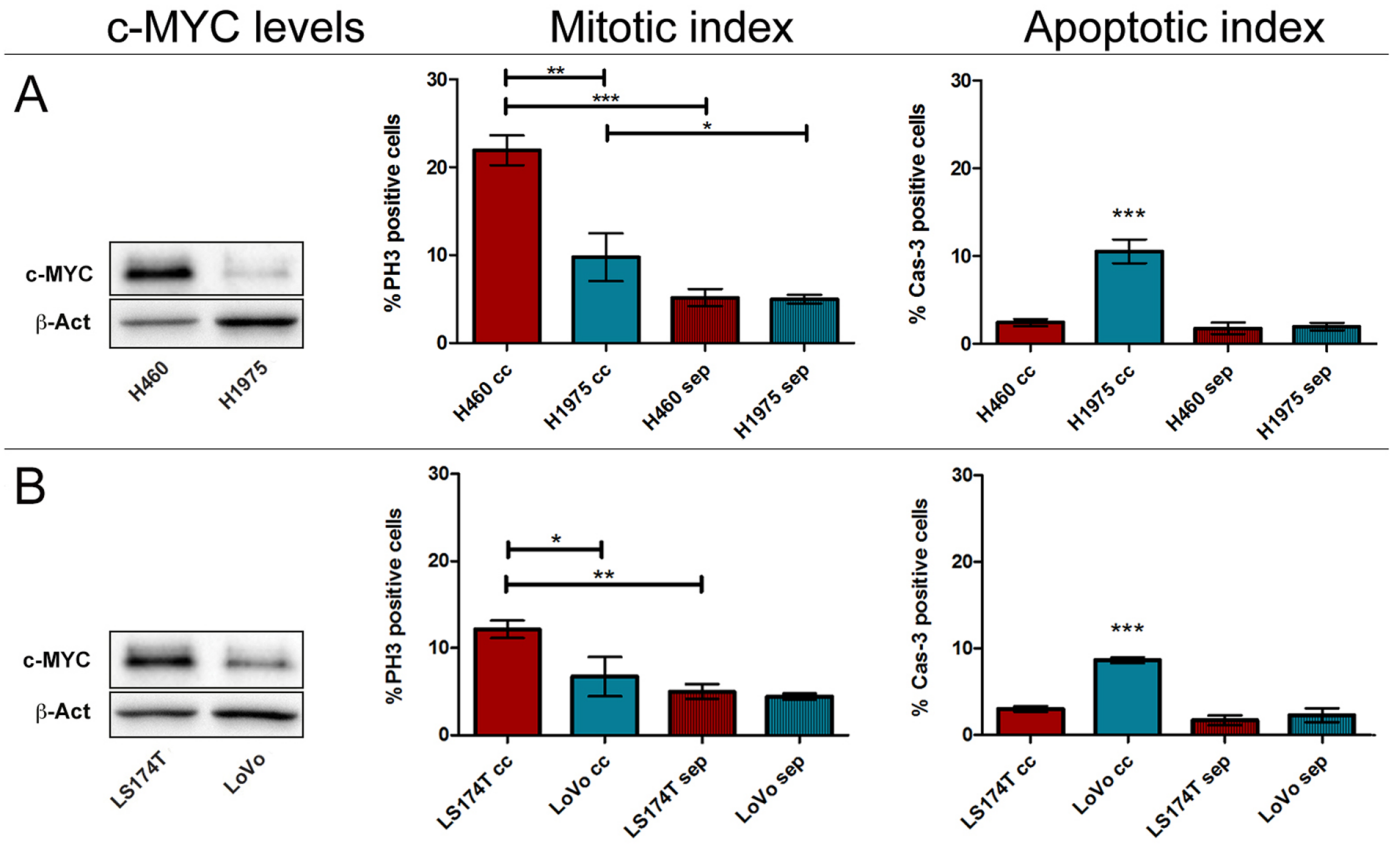
Human cancer cell lines displaying different c-MYC protein levels show competitive behaviours in co-culture. (**A**) H460 *vs* H1975 lung cancer cell lines. (**B**) LS174T *vs* LoVo colon cancer cell lines. In each assay, the Western Blotting shows c-MYC protein levels of the two cell lines used and β-actin as a loading control. The Mitotic Index shows the percentage of PH3-positive cells in the two lines after 5 h in co-culture (cc) or separate (sep) conditions; the Apoptotic Index is represented as the percentage of Cas3-positive cells at the end of the assay. Original blots are presented in Supplementary Figure S7. Statistical significance and ± SD are indicated.

**Figure 7:**
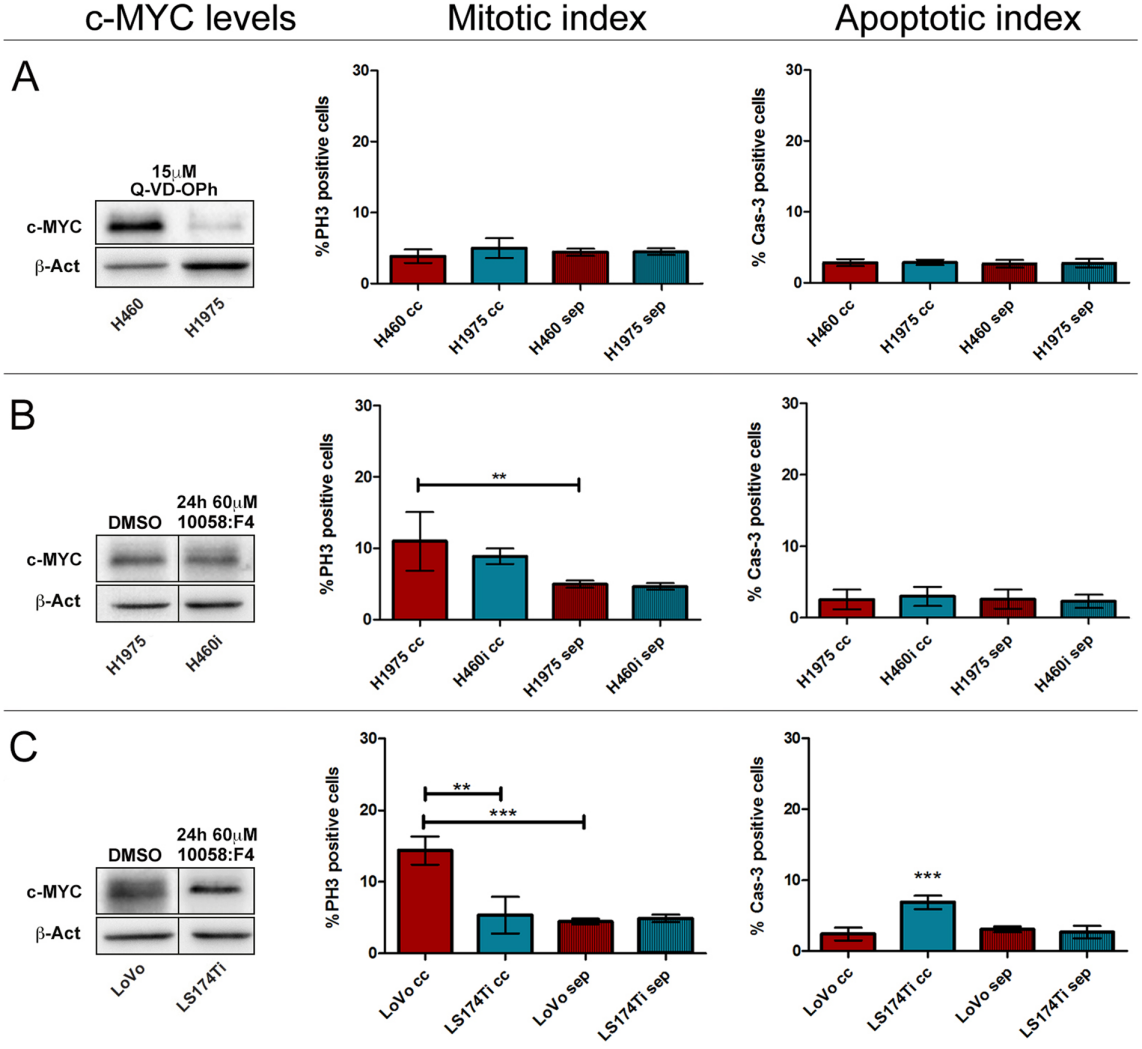
Apoptosis and MYC inhibition are both sufficient to impair the competitive ability of human cancer cell lines. (**A**) H460 *vs* H1975 lung cancer cell lines treated with the pan-caspase inhibitor Q-VD-OPh. (**B**) H1975 *vs* H460i (inhibited) lung cancer cell lines. (**C**) LoVo *vs* LS174Ti (inhibited) colon cancer cell lines. In each assay, the Western Blotting shows c-MYC protein levels of the two cell lines used and β-actin as a loading control (crops are indicated by black lines, the original blots can be found in Supplementary Fig. S7), with treatments indicated at the top of the lanes; the Mitotic Index shows the percentage of PH3-positive cells in the two lines after 5 h in co-culture (cc) or separate (sep) conditions; the Apoptotic Index is represented as the percentage of Cas3-positive cells at the end of the assay. Statistical significance and ± SD are indicated.

As many, if not all, genetic alterations recurrent in epithelial malignancies impinge on c-MYC activity^51^, it is conceivable that neighbouring cells compare the relative c-MYC levels as a “readout” of their overall metabolic and genetic condition. By simplification, competition in cancer would be dictated by the global molecular changes of the cell, with c-MYC activity representing their algebraic sum. Changes in c-MYC expression levels do not indeed account on gene mutation^52^, therefore they may represent a plastic marker of cell fitness. To support this hypothesis, we developed a co-culture assay (CCA) similar to those used by other groups to ascertain MMCC function in several cell types^7,8,15,21,53^. In this case, we co-cultured pairs of different human cancer cell lines, thus reproducing a condition of genetic heterogeneity in the plate. In addition, since it is known that MYC induction primes immediate death of the confronting population in co-cultures of *Drosophila* S2 cells^53^, we limited our assays to 5 hours, after which we measured mitotic and apoptotic indexes of both cell lines, either in co-culture or in separate conditions. A set of two human lung cancer lines, H460 and H1975, and a set of two human colon cancer lines, LS174T and LoVo, were used in the assays. Each pair presented different c-MYC protein levels (the original blots are shown in Supplementary Fig. S7A). The results of the CCAs are displayed in Fig. 6, where each panel is composed of a Western Blotting (WB), showing the c-MYC levels of the two cell lines used in the assay, and two graphs showing the mitotic and the apoptotic indexes at the end of the assay (5 h from seeding). The mitotic index was measured as the percentage of Phospho-Histone H3 (PH3)-positive cells, and the apoptotic index was measured as the percentage of activated Caspase 3 (Cas3)-positive cells (see Supplementary Fig. S8 for representative photographs). Both measures were taken for each cell line grown in co-culture (cc) and in separate conditions (sep). Cell populations participating in each assay were alternatively labelled with a fluorescent dye to distinguish them during and after the assay (See Methods and Supplementary Figure S9A,B). In all the CCAs, red bars represent the winner cells and blue bars represent the loser cells. As can be observed in Fig. 6A, c-MYC levels in the H460 cells were higher than those showed by the H1975 line. After the CCA, while both H460 and H1975 lines showed a comparable mitotic index in separate conditions, they displayed quite different mitotic indexes following co-culture (compare sep and cc in Fig. 6A). The proliferation rate of the H460 cells in co-culture was significantly higher both respect to the H1975 partner line and respect to the H460 cells grown in separate conditions. The growth units at 0 and 5 h from seeding are given in Supplementary Fig. S10A. The growth disadvantage of the H1975 cells in co-culture was associated with an increase in apoptotic cell death: the percentage of Cas3-positive cells was indeed significantly higher in the H1975 cc cells than in all the other samples. Of note, the mitotic index of the H1975 loser cell line was higher in cc than in separate conditions, indicating that, such as it happens in *Drosophila*, dying cells may emit pro-growth factors that, further to be exploited by winner cells, may be used by competent prospective losers to proliferate and escape untimely death^54^. Similar behaviours have indeed been described in mammalian cancer models^55,56^. The same assay was repeated using the two colon cancer lines, LS174T and LoVo. As can be appreciated in the WB, the LS174T cell line showed higher c-MYC levels than the LoVo partner line (Fig. 6B), and it was accordingly found to out-compete the LoVo cells in the assay, which indeed showed the highest percentage of Cas3-positive cells. Also in this case, the proliferation rate of the winner cells in co-culture was significantly higher both respect to the LoVo partner cells and respect to the LS174T cells grown in separate conditions (Fig. 6B and Supplementary Fig. S10B). We next assessed if apoptosis was necessary for MMCC to occur, as it is *in vivo* in *Drosophila* ^5,6,57^; we thus repeated the CCA between the H460 and H1975 lung cancer lines in the presence of a pan-caspase inhibitor (see Methods and Supplementary Fig. S9C), and we found that competitive interactions were completely abrogated. No differences in terms of proliferation or apoptotic death were indeed observed (Fig. 7A and Supplementary Fig. S10C). As we were interested in analysing the effect of MYC down-regulation on the winners’ competitive behaviour, we tested the 10058:F4 MYC specific inhibitor^58^ (see Methods and Supplementary Fig. S9A,B and S11 for validation) in all the cell lines used in the assays. Supplementary Figures S12 and S10F-I show that MYC inhibition in isogenic backgrounds was sufficient to turn all the lines to losers following confrontation with the parental line in native conditions, confirming what was previously obtained through MYC knockdown^21^. We then repeated the same CCAs as in Fig. 6 following MYC inhibition in the winner lines. As can be seen in Fig. 7B, c-MYC protein levels in the H460 and H1975 lines result comparable after MYC inhibition in the H460 cells (H460i) (the original blots of treated and untreated lines are shown in Supplementary Fig. S7B), and the two lines no longer compete when placed in co-culture, showing comparable mitotic and apoptotic indexes as those observed in separate conditions (Fig. 7B and Supplementary Fig. S10D). We then repeated the same assay using the LS174T and LoVo cell lines. In Fig. 7C the WB shows that c-MYC inhibition in the LS174T line is sufficient to lower protein levels below those displayed by the LoVo line, and the CCA highlights that this latter acquires the condition of winner, with a mitotic index significantly higher compared to that of the loser line and of the LoVo line grown in separate conditions. On the other hand, the LS174T reversed its condition from winner to loser, displaying the highest percentage of Cas3-positive cells (Fig. 7C and Supplementary Fig. 10E). Despite the cells in co-culture experienced the presence of different neighbours for just five hours, we moreover noticed that, in some cases, the final cell count was amazingly different, either between the winner and the loser cells in co-culture or between the same lines grown in co-culture and in separate conditions (Supplementary Figure S10). Altogether, our findings suggest that changes in MYC activity during cancer progression may drive competitive clonal selection downstream of genetic heterogeneity.

## Discussion

Tumours undergo continuous genetic diversification and epigenetic plasticity, while selection favours mutations that increase cell’s ability to survive in the changing context^59^. In this dynamic scenario, MYC-Mediated Cell Competition (MMCC) may emerge as a cancer trait priming clonal selection and expansion, thus contributing to the final cell composition and mass dimensions. Given the striking evolutionary conservation of this mechanism in development, we explored the possibility to find stereotypical patterns of MMCC in human cancers. As reported in Figs 1, 2 and 3, tumours displaying polarity loss and deregulation of the Hippo pathway show a number of stromal cells at the tumour interface positive to Caspase 3 staining. A quantification of Cas3 and c-MYC signals revealed that cell death of stromal cells was highly correlated to c-MYC protein levels in the tumour cells, regardless of tumour stage and type, suggesting that MMCC may work between different cell histotypes. Of note, fibroblasts have been demonstrated to be sensitive to MYC levels in cell competition assays^15^ and, such as it happens in *Drosophila* ^60^, some cells with phagocytic function recruited to the tumour site may also engulf dying cells, forming the clusters of Cas3- and TUNEL-positive cells observed (as an example, see Fig. 1F,L). We also found groups of cancer cells dying when surrounded or adjacent to high MYC-expressing cells (Fig. 5). Considering the high genetic divergence of cells composing advanced malignancies, this observation suggested that MYC levels may signal cellular performance downstream of genetic aberrations. To support this hypothesis, we co-cultured pairs of different human cancer cell lines demonstrating that, whatever the genotype of the cells, MYC protein modulation was sufficient to subvert their competitive behaviour (Fig. 7B,C). MYC activity in cancer cells may thus represent a plastic readout of their aberrant gene expression, on the basis of which cells measure their ability to compete in the changing context. Our findings highlight the importance of developing new, interdisciplinary cancer models where to investigate how cell competition shapes cancer evolution, so to conceive novel therapeutic interventions that may help “good” cells out-compete the “bad” ones.

## Methods

### Immunohistochemistry (IHC) on human samples

IHC staining of tumour samples was carried out on 3μm formalin-fixed paraffin-embedded (FFPE) sections. YAP staining (rabbit polyclonal antibody, Cell Signaling Technology #4912, 1:150)^61^ and HUGL-1 staining (rabbit polyclonal antibody, D. Strand, 1:550)^37^ were performed on consecutive slices using UltraVision Kit (Thermo Scientific) and the DAB Chromogen System (Dako). c-MYC staining (mouse monoclonal antibody 9E10, DSHB, 1:50)^62^ and Cleaved-Caspase-3 staining (rabbit polyclonal antibody, #9961 Cell Signaling Technology, 1:250)^63^ were performed on the same slice using the EnVision G/2 Doublestain System (Dako). Cell nuclei were counterstained with Mayer’s Hematoxylin. Slides were scanned using an Aperio Scanscope CS System by Leica Biosystems and original digital frames were used to compose the figure panels. Samples were from the Bellaria Hospital archives (Bologna, Italy), where informed consent was obtained prior to sample banking. Institutional Review Board approval (Nr. 20OS11) was obtained at the Bologna AUSL (Azienda Unità Sanitaria Locale). All experiments were performed in accordance with relevant guidelines and regulations.

### TUNEL assay on IHC samples

TUNEL assays were performed using the “*In situ* cell death detection kit”, POD (Ct. 11684817910, Roche Applied Sciences) following the manufacturer’s protocol for 3-µm FFPE sections, with minor modifications. Briefly, sections were dewaxed for 90 min at 65 °C, 20 µg/ml proteinase K solution (Cat. No 000000003115844001- Roche Applied Sciences) was applied for 15 min RT and stopped by two fast PBS1X washing steps; the slides were then immersed in 3% BSA-PBS 1X blocking solution for 30 min RT. The sections were washed and incubated with the TUNEL Reaction Mixture for 60 min at 37 °C in a moist room. After two washing steps, 3% BSA-PBS 1X blocking solution was applied again for 30 min RT, followed by two washing steps. Subsequently, the tissues were incubated with a 1:3 solution of converter-POD in 1% BSA-PBS1X for 30 min at 37 °C. Sections were finally stained with DAB and counterstained with Mayer’s Hematoxylin.

### IHC image analysis

With regard to the single stainings for HUGL-1 or YAP, we used the “Color deconvolution” plugin in ImageJ to deconvolve IHC RGB images into separate channels for Hematoxylin and the chromogen DAB. For the double staining Cas3/c-MYC, the same software was used to deconvolve RBG images into separate channels for Hematoxylin, DAB (chromogen for Cas3) and Permanent Red (chromogen for c-MYC). Cas3 stainings were then subdivided into three classes scored 0 (no signal), 1 (medium signal) and 2 (high signal), and 10 fields/tumour sample were quantified. Each tumour sample was then given a mean score by averaging those of the 10 fields analysed. Quantification of c-MYC staining was obtained by measuring the reciprocal mean intensity of a representative ROI in each frame as above. Unlike immunofluorescence (IF) samples, in which the brightness of a region is directly proportional to the amount of antigen, chromogen stains appear darker in regions with more antigen. We therefore calculated the “reciprocal” intensity by subtracting the intensity of the stained ROI from the maximum value^64^.

### The basics of cell competition assay (CCA)

Each experiment was carried out in two simultaneous technical replicates using 6-well plates filled as follows: wells 1–2: co-cultures (2.5 × 10^5^ cells from each population); wells 3–4 and 5–6: separate controls (5 × 10^5^ cells). After 5 hours incubation cells were detached and counted. The growth units for each assay (see Supplementary Fig. S10) were calculated as the ratio: final cells/initial cells. For Caspase 3 and Phospho-Histone 3 staining, cells were seeded on a coverslip. For each assay, from three to four experimental replicates were carried out.

### Immunofluorescence on human cells

IF staining of human cells grown on coated coverslips was performed according to standard protocols. For each sample, four fields containing at least 100 cells were captured under a confocal microscope and counted (representative fields can be found in Supplementary Fig. S8). The primary antibodies were rabbit α-Phospho-Histone H3 (Upstate, pSer 10, 1.100) and α-cleaved Caspase 3 (#9961 Cell Signaling, 1:100), and the secondary antibody was α-rabbit, 555 Alexa Fluor 1:400. Cell nuclei were counterstained with DAPI.

### Cell culture

Human cancer cell lines - lung NCI-H460 (HTB-177) and NCI-H1975 (CRL-5908); colon LS174T (CL-188) and LoVo (CCL-229) - were cultured in DMEM under standard conditions. Cell lines were tested for c-MYC levels by WB before each assay.

### Western blotting analysis

Total cellular protein extracts were obtained and normalised by standard procedures. Detection was performed with HRP-ECL (Bio-Rad). Primary antibodies were rabbit α-c-MYC (Santa Cruz, N-262; 1:1500)^65^ and rabbit α-β-Actin (Jackson ImmunoResearch; 1:2000). The HRP-conjugated secondary antibodies were from Jackson ImmunoResearch, 1:2000.

### Cell membrane labelling

To discriminate co-cultured cell populations, one of the two was labelled with 10μM PKH67 fluorescent dye (Sigma-Aldrich) prior to assay according to the manufacturer’s protocol.

### Chemical treatments

10058:F4 (Sigma-Aldrich)^58,66,67^ was used at 60 µM to drop c-MYC expression prior to competition assay (Supplementary Fig. S11A). This concentration was chosen among 40 µM, 60 µM and 80 µM as it gave appreciable results in inhibiting both c-MYC expression and transcriptional activity (Supplementary Fig. S11A,B) without affecting native indexes of cell proliferation and cell death (Supplementary Fig. S9A,B). After 24 hours treatment, cells were washed and seeded as above described (The basics of cell competition assay). c-MYC transcript and protein levels were verified before and after the 5 hours assay (Supplementary Fig. S11). The pan-caspase inhibitor Q-VD-Oph (Sigma-Aldrich) was added to co-cultured and separate cells at 15 µM (see Supplementary Fig. S9C).

### Statistical analysis

The Pearson correlation coefficient and the respective *p*-value were calculated for each tumour type by comparing Cas3 and c-MYC scores attributed to each sample (Supplementary Table S1, column E). For CCAs, statistical significance was determined by using two-tailed, unpaired *t-*tests from at least three independent replicates and expressed as *p* values: *p* ≤ 0.05 = *, *p* ≤ 0.01 = **, *p* ≤ 0.001 = ***. Results are presented as mean s.d. in bar graphs created in GraphPad Prism 5.

### Data availability

No datasets were generated or analysed during the current study.

## Electronic supplementary material

Supplementary Information
