# Supplementary material for: Human Cancer Cells Signal Their Competitive Fitness Through MYC Activity
Source: Sci Rep. 2017 Oct 3;7:12568. doi: 10.1038/s41598-017-13002-1 (PMC5626713; doi:10.1038/s41598-017-13002-1)
Supplement: Supplementary file 1 — Supplementary Information [file 41598_2017_13002_MOESM1_ESM.pdf]

Human Cancer Cells Signal Their Competitive Fitness Through MYC Activity

Simone Di Giacomo, Manuela Sollazzo, Dario de Biase, Moira Ragazzi, Paola Bellosta, Annalisa Pession, Daniela Grifoni

SUPPLEMENTARY INFORMATION

Di Giacomo et al. Supplementary Table S1.

**A**<br><br>**Tumour type**	**B**<br><br>**Sample number**	**C**<br><br>**Caspase mean score**	**D**<br><br>**c-MYC mean score**	**E**<br><br>**Pearson correlation coefficient**
**Colon**	9	0.8<br>1.5<br>1.2<br>1.4<br>1.2<br>1.1<br>1.3<br>0.9<br>1.3	54<br>168<br>123<br>168<br>119<br>92<br>152<br>65<br>159	0.9743<br><br>$p < 0.001$
**Breast**	10	1.7<br>1.5<br>1.4<br>1.5<br>1.5<br>1.7<br>1.6<br>1.6<br>1.9<br>1.6	218<br>187<br>171<br>189<br>179<br>210<br>199<br>198<br>239<br>201	0.986<br><br>$p < 0.001$
**Lung**	8	1.3<br>1.6<br>1.5<br>1.4<br>1.4<br>1.6<br>1.8<br>1.4	148<br>202<br>182<br>165<br>159<br>196<br>227<br>171	0.989<br><br>$p < 0.001$

Supplementary Table S1. Correlation between c-MYC and Caspase 3 levels in tumour samples. The table reports the cases analysed in the study subdivided according to tumour type, with the respective Caspase and c-MYC scores. For each sample, caspase score represents the mean of individual values (absent/low=0; medium=1; high=2, see main Figure 4) obtained from 10 fields containing 50-75% tumour cells. c-MYC scores represent the mean of individual red values in the same fields as above (see Figure S4 for details). The Pearson correlation coefficient and its statistical significance are indicated for each tumour type.

Di Giacomo et al. Supplementary Figure S1.

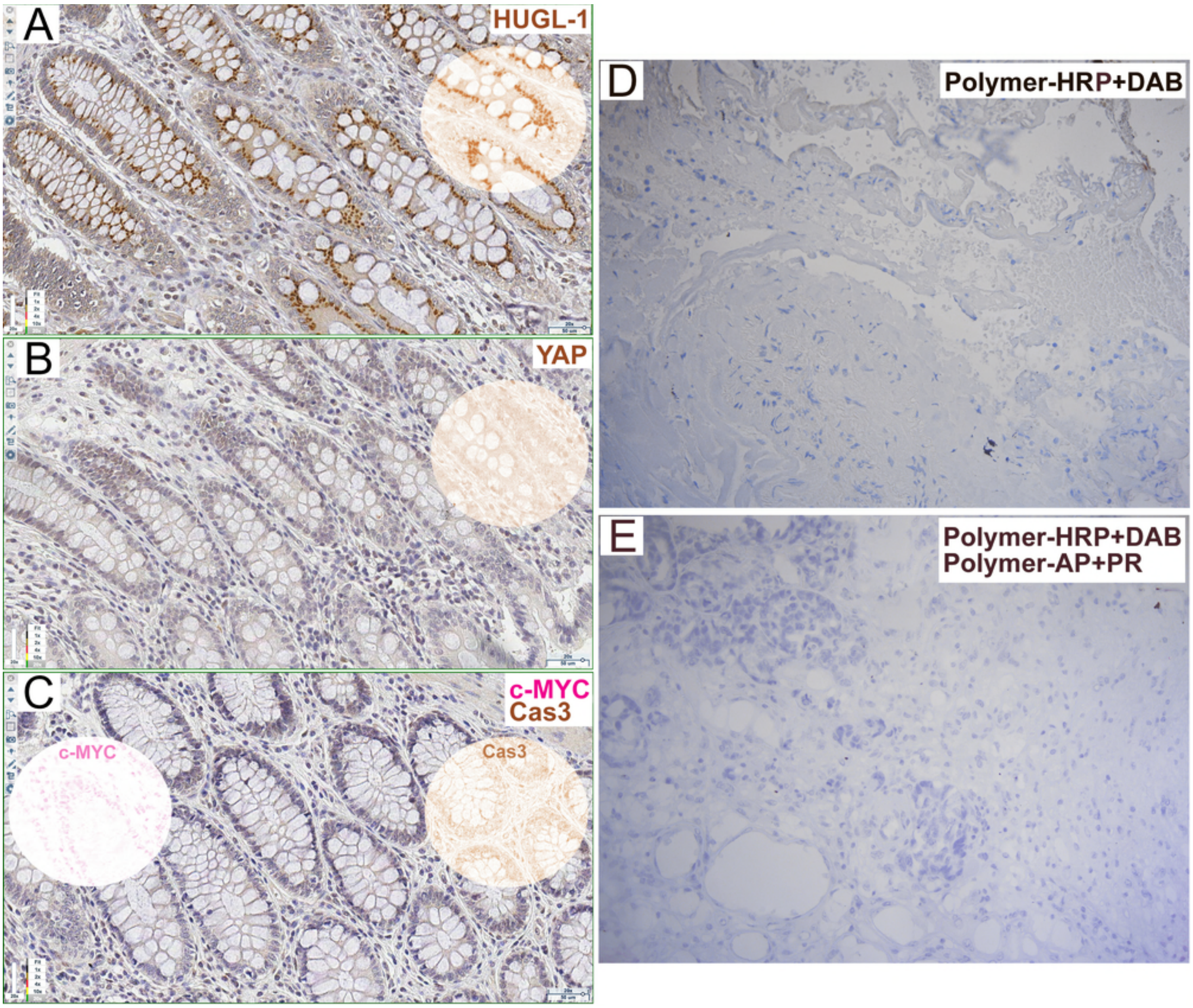

Supplementary Figure S1. HUGL-1, YAP, c-MYC and Cas3 staining of normal tissue. **A-C**, normal colon stained for HUGL-1 (brown) (**A**), YAP (brown) (**B**) and c-MYC (red) and Cas3 (brown) (**C**). Insets show the same IHC stainings deconvolved from RGB into single DAB (HUGL-1, YAP and Cas3) and PR (c-MYC) channels. (**D-E**) single (HUGL-1 and YAP) and double (c-MYC+Cas3) staining controls: breast cancer tissue stained with an HRP-conjugated polymer and its substrate DAB (3,3'-diaminobenzidine) (**D**) and an HRP-conjugated polymer and its substrate DAB plus an AP-conjugated polymer and its substrate PR (Permanent Red) (**E**).

Di Giacomo et al. Supplementary Figure S2.

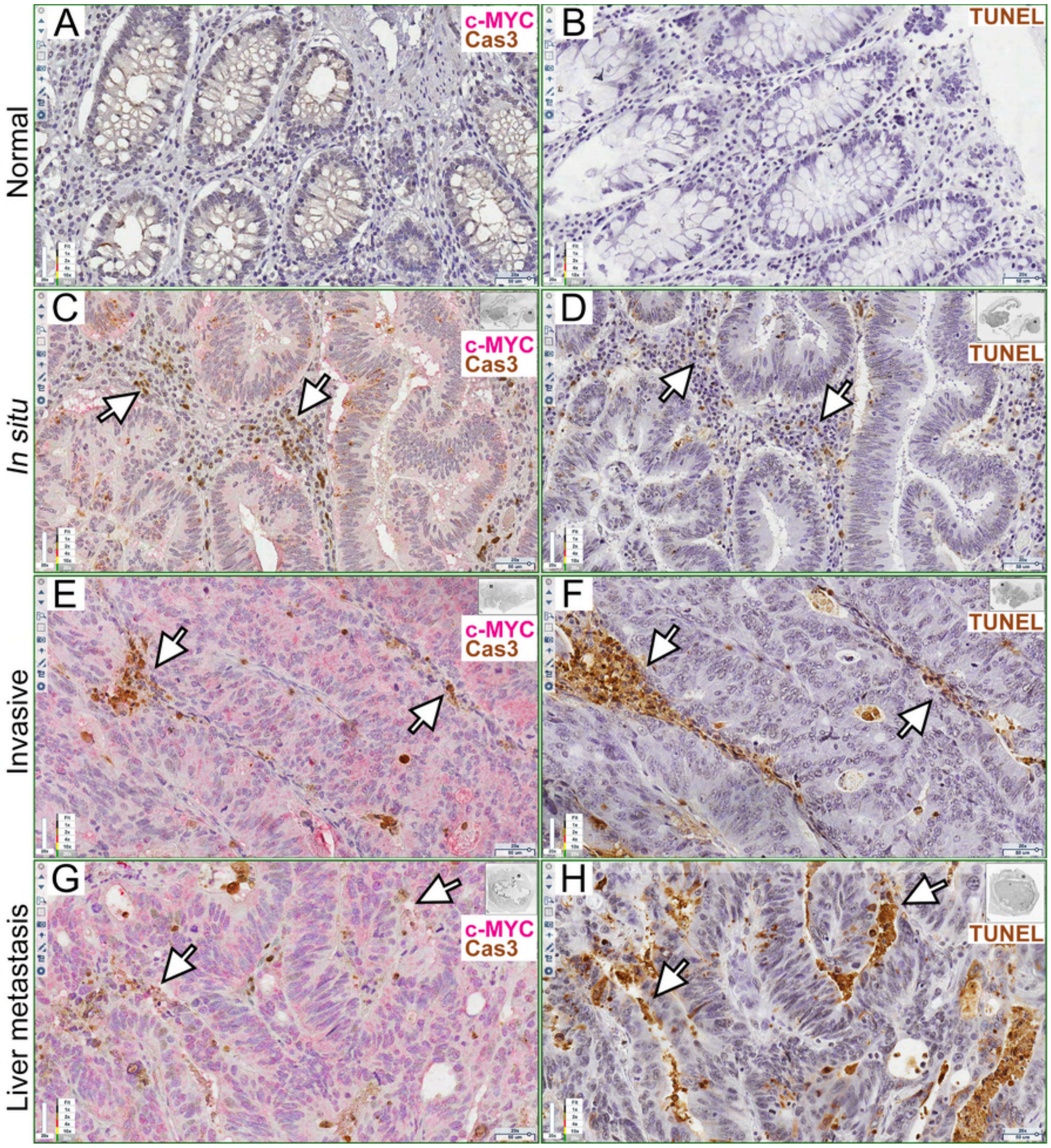

Supplementary Figure S2. TUNEL assay performed on normal colon tissue and colon cancers. **A-B**, normal colon stained for c-MYC (red) and Cas3 (brown) (**A**) and TUNEL (brown) (**B**). **C-H**, sequential slices of colon cancers at different stages of the disease stained for c-MYC (red) and Cas3 (brown) (**C**,**E**,**G**) and TUNEL (brown) (**D**,**F**,**H**). The ROI can be found in the thumbnail at the upper-right of each frame, and the scale bars are included in the pictures. The arrows indicate some examples of cells positive to both Cas3- and TUNEL.

Di Giacomo et al. Supplementary Figure S3.

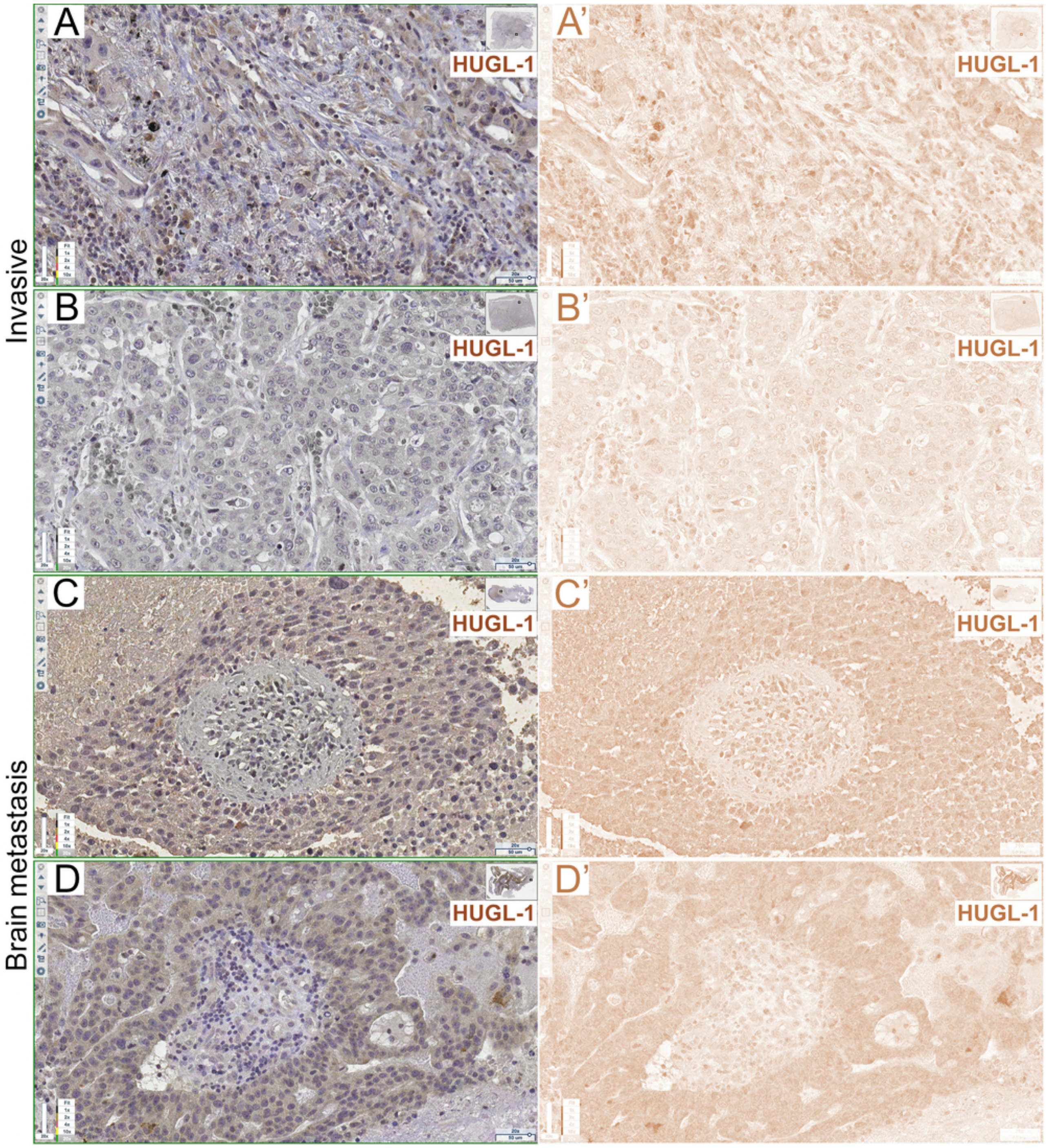

Supplementary Figure S3. HUGL-1 abundance and localisation in tumour samples. **A-D,** lung cancer RGB images from main Figure 3 that underwent colour deconvolution. **A'-D'**, DAB channel (HUGL-1).

Di Giacomo et al. Supplementary Figure S4.

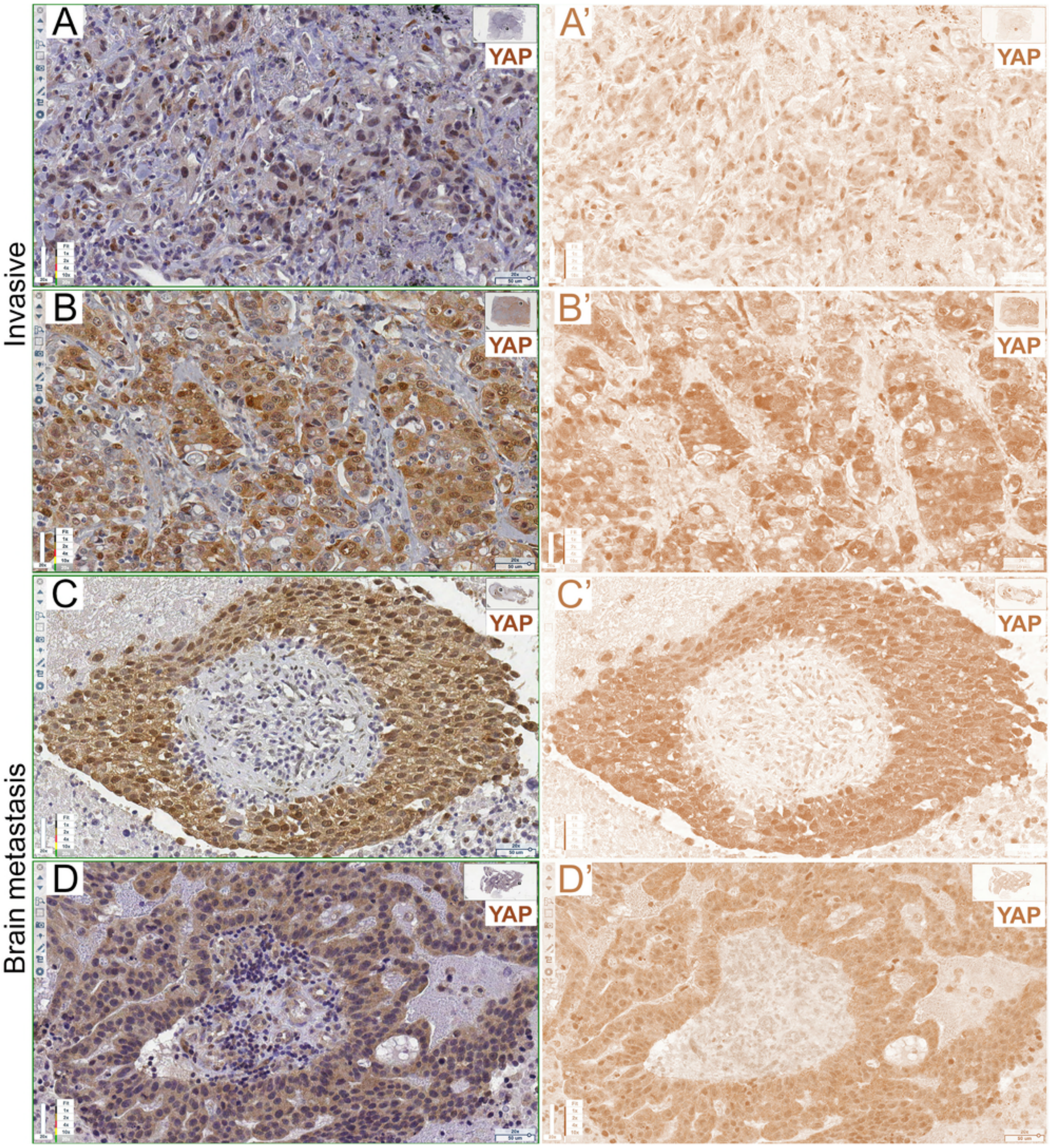

Supplementary Figure S4. YAP abundance and localisation in tumour samples. **A-D,** lung cancer RGB images from main Figure 3 that underwent colour deconvolution. **A'-D'**, DAB channel (YAP).

Di Giacomo et al. Supplementary Figure S5.

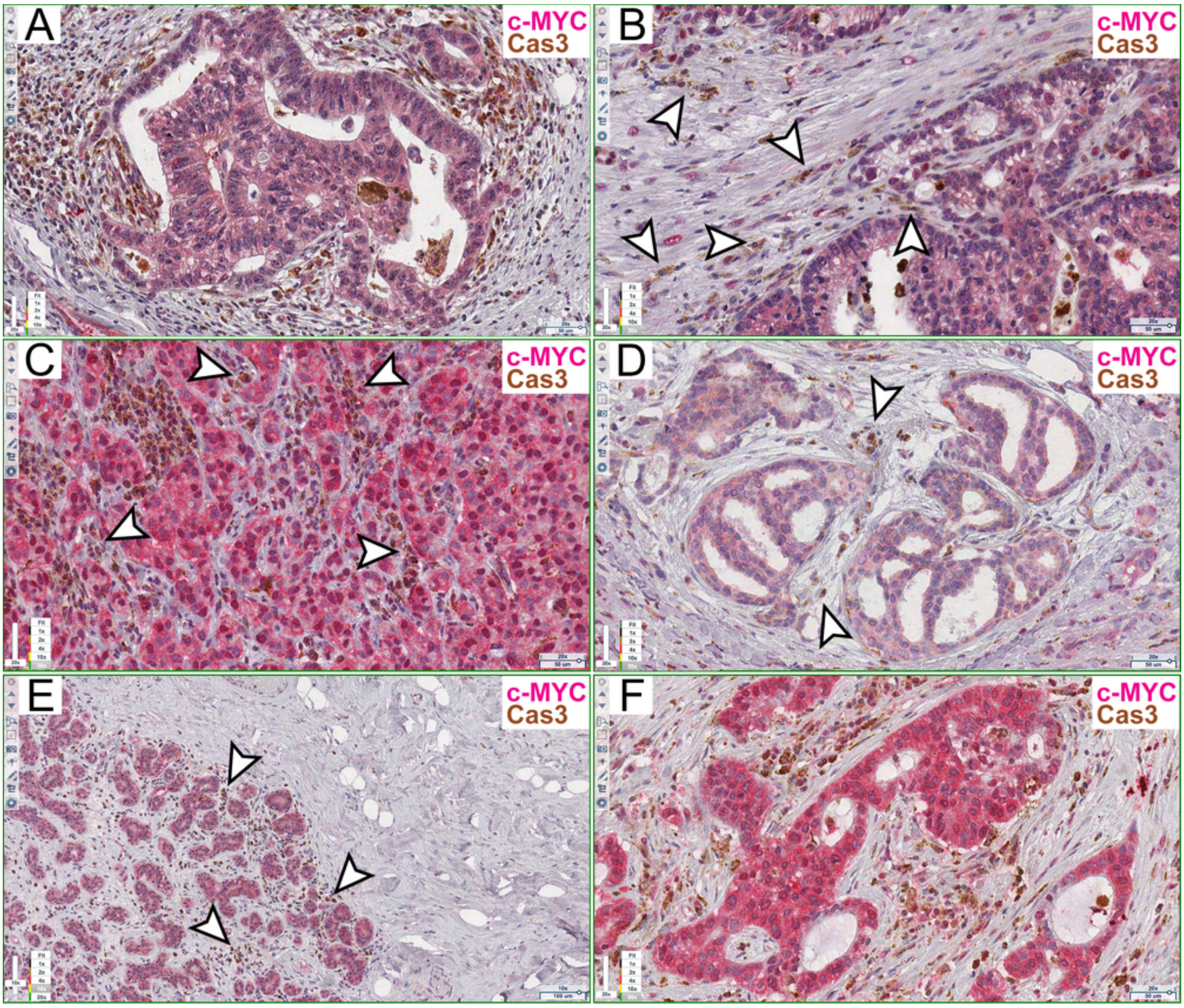

Supplementary Figure S5. Additional examples of cell death at the tumour-stroma interface. Colon cancers (**A**,**B**), breast cancers (**C**-**E**) and lung cancer (**F**) stained for c-MYC (red) and Cas3 (brown). The arrowheads indicate some Cas3-positive cells in **B**-**E**. In **A** and **F** cell death is self-evident. The scale bars are included in the pictures.

Di Giacomo et al. Supplementary Figure S6.

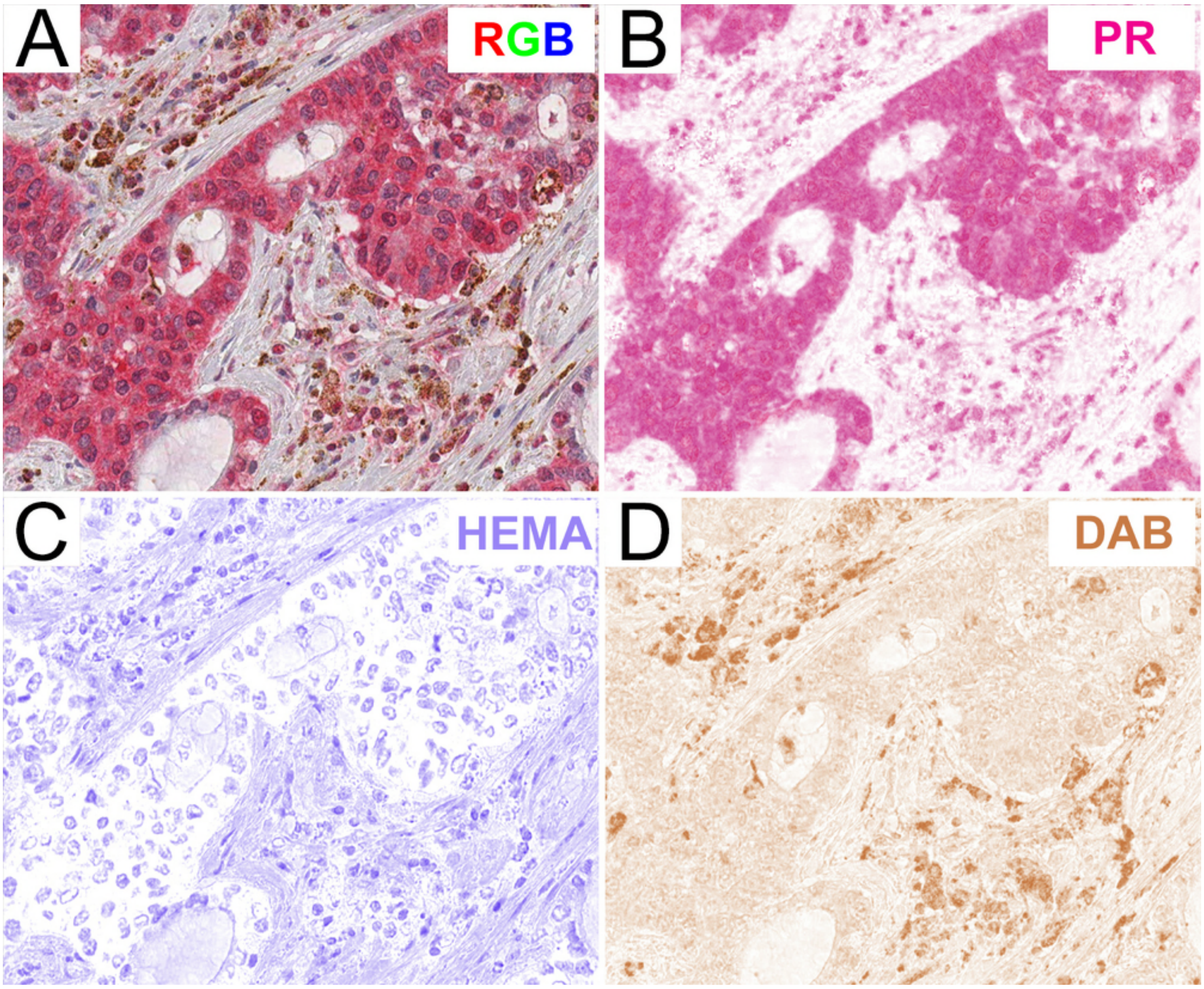

Supplementary Figure S6. Quantification of c-MYC abundance in tumour samples. **A,** an example of an RGB image that underwent colour separation through the "*Colour deconvolution*" plugin for ImageJ (NIH, Bethesda). **B**, Permanent Red channel (c-MYC); **C**, Hematoxylin channel (cell nuclei); **D**, DAB channel (Cas3). Full details are given in the Methods section.

Di Giacomo et al. Supplementary Figure S7.

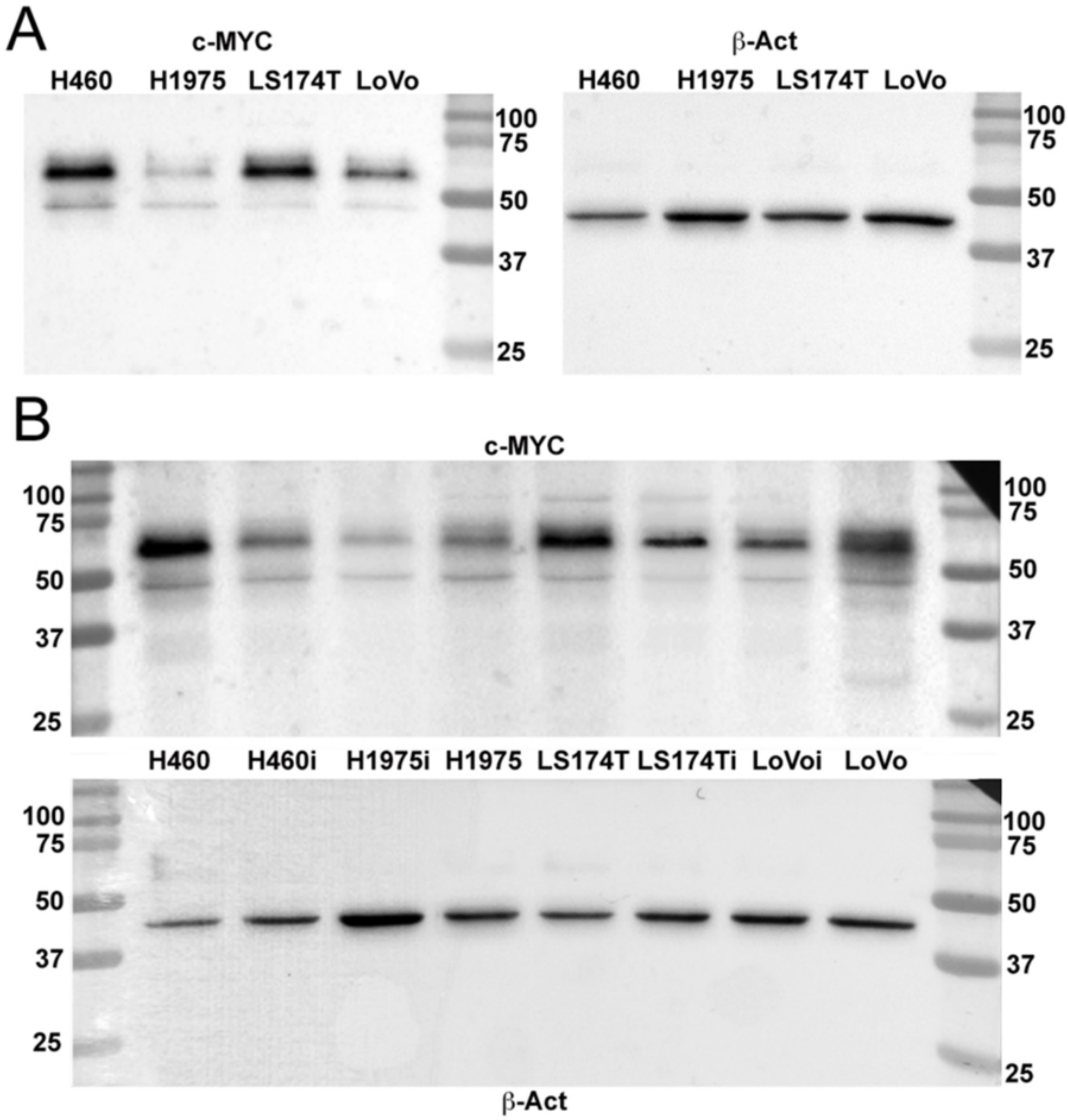

Supplementary Figure S7. Original Western Blotting membranes showing c-MYC protein levels in untreated and treated human cell lines used in this work. **A,** c-MYC protein levels in untreated cell lines, β-actin is shown as a loading control. **B,** c-MYC protein levels in pairs of untreated (DMSO) and treated (60μM 10058:F4) cell lines, β-actin is shown as a loading control. The molecular weight marker is also shown ("Precision Plus Protein Dual Color Standards" from Bio-Rad).

Di Giacomo et al. Supplementary Figure S8.

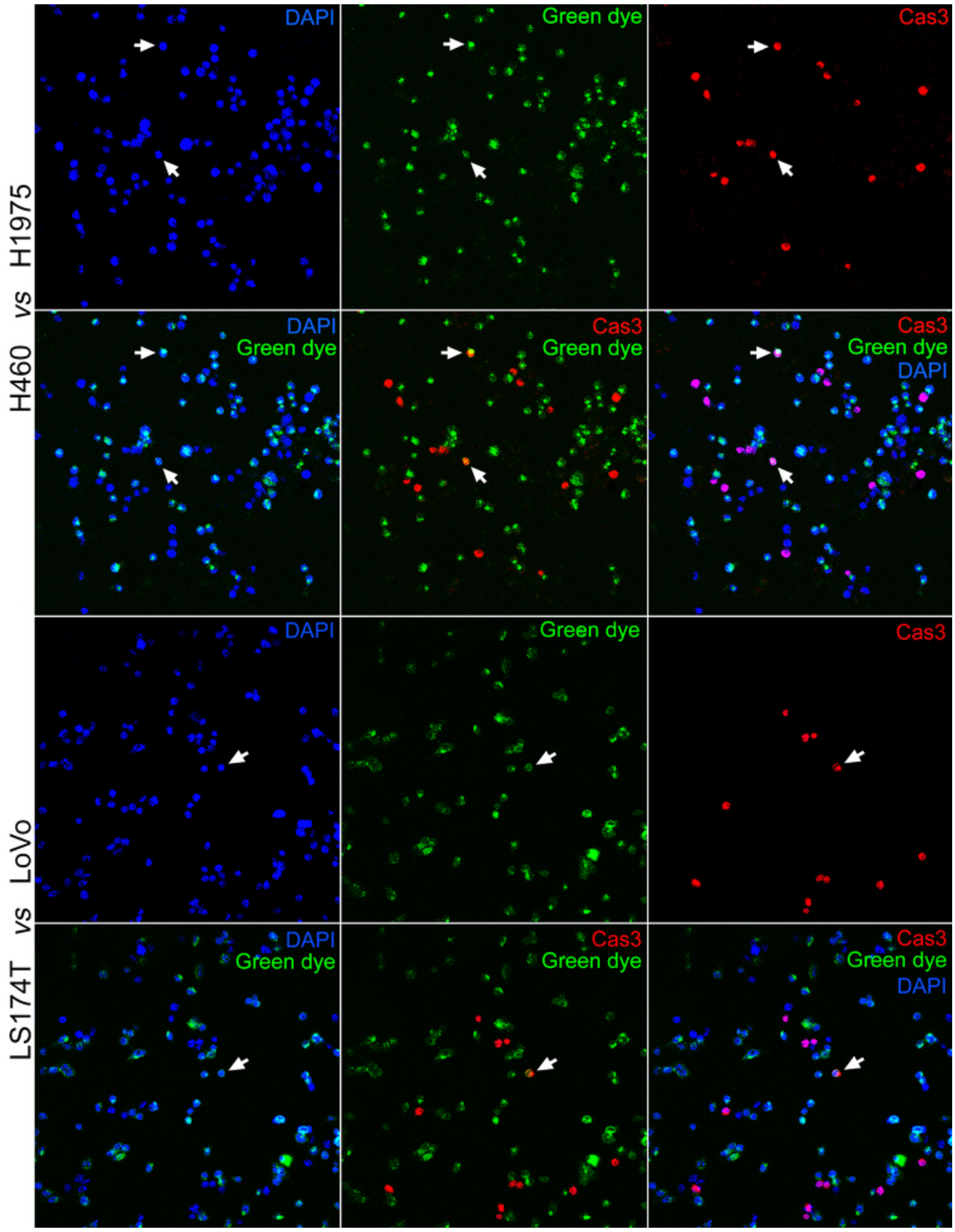

Supplementary Figure S8. Representative photographs used to count Cas3-positive cells after the CCAs. The upper panel shows a 200X field of a CCA performed on native H460/H1975 lung cancer lines, while the lower panel displays a 200X field of a CCA performed on native LS174T and LoVo colon cancer lines. DAPI stains cell nuclei, the green dye marks in this case the prospective winner cells and Cas3 identifies the dying cells. The arrows indicate Cas3-positive winner cells in the fields.

Di Giacomo et al. Supplementary Figure S9.

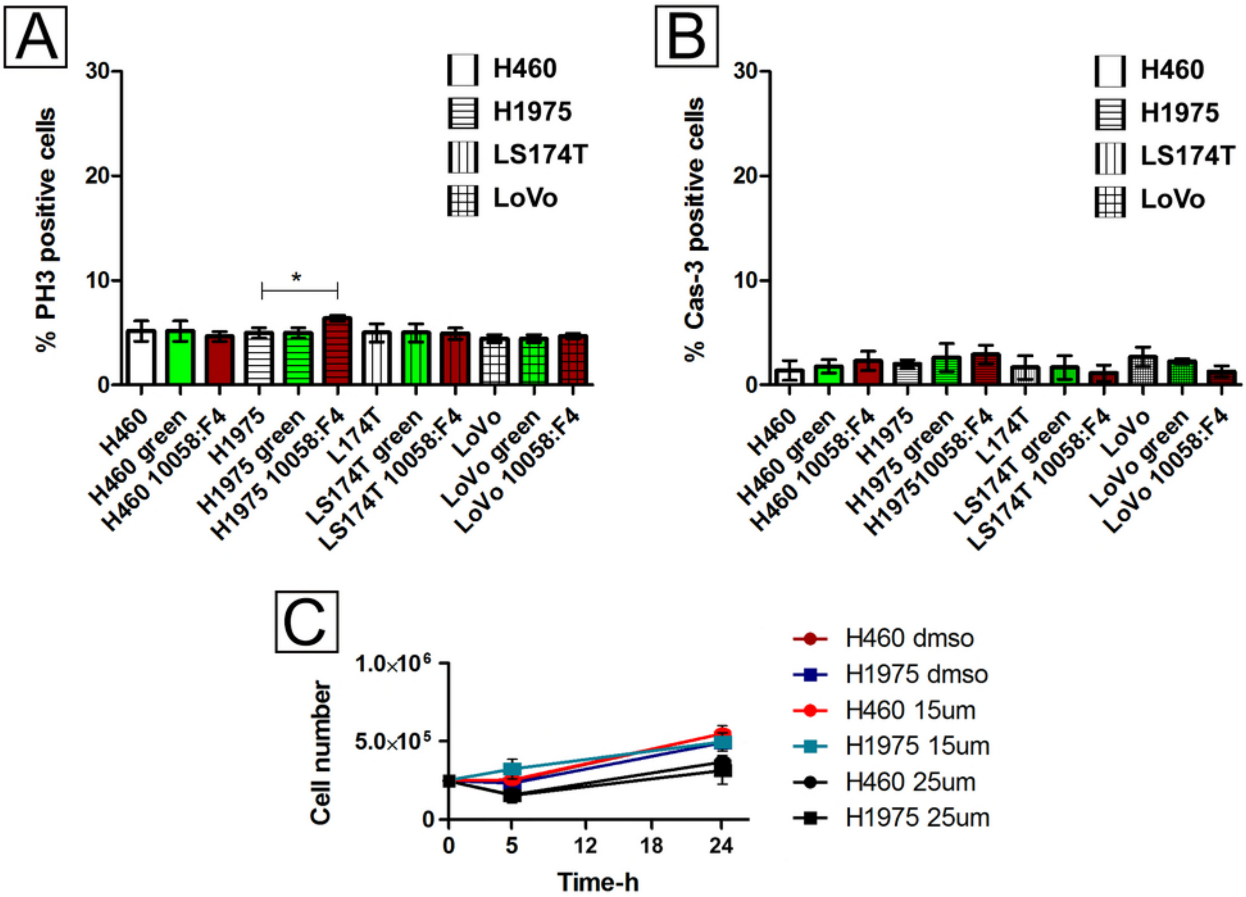

Supplementary Figure S9. Impact of different treatments on the mitotic and apoptotic indexes of the cell lines used in the study. **A**, mitotic indexes of the cell lines grown in separate conditions at 5h from seeding. Plain white=no treatment; plain green=fluorescent dye; plain red=c-MYC inhibitor. **B**, apoptotic indexes of the cell lines grown in separate conditions at 5h from seeding. Plain white=no treatment; plain green=fluorescent dye; plain red=c-MYC inhibitor. Statistical significance and SD are indicated as an average from four independent replicates. *p*-values are: $p<0.05$ (*), $p<0.01$ (**) and $p<0.001$ (***). **C**, proliferation indexes of the cell lines used in the Caspase inhibition assay (main Figure 7). Cells were harvested and counted at 5 and 24 hours from seeding.

Di Giacomo et al. Supplementary Figure S10.

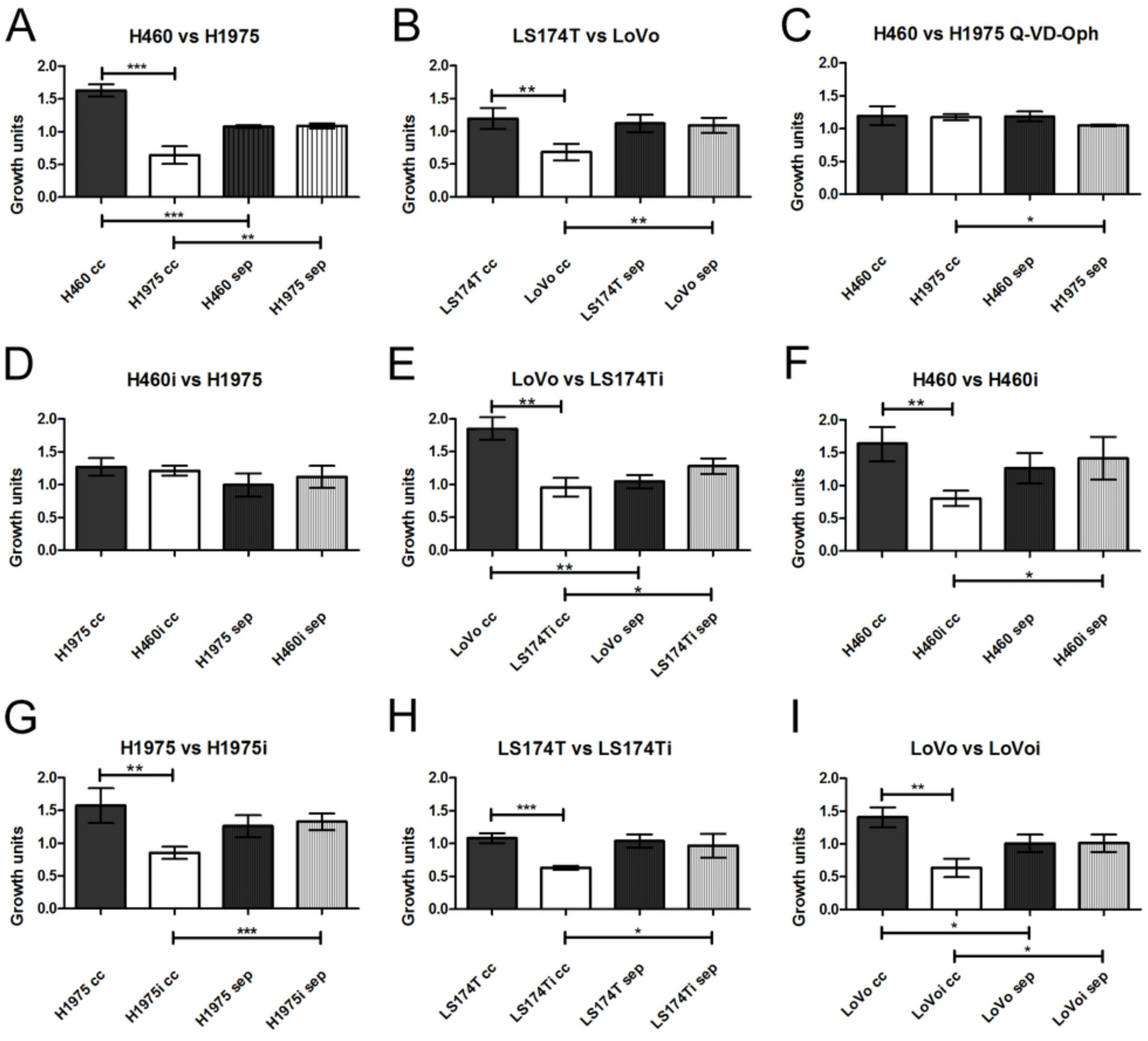

Supplementary Figure S10. Growth units of the different cell lines after 5-hours CCAs. The graphs are in the same order as described in the text. At time 0, the Growth unit value is 1. For each experiment, the average value of at least three independent replicates is shown with the respective SD. *p*-values are: $p<0.05$ (*), $p<0.01$ (**) and $p<0.001$ (***).

Di Giacomo et al. Supplementary Figure S11.

A

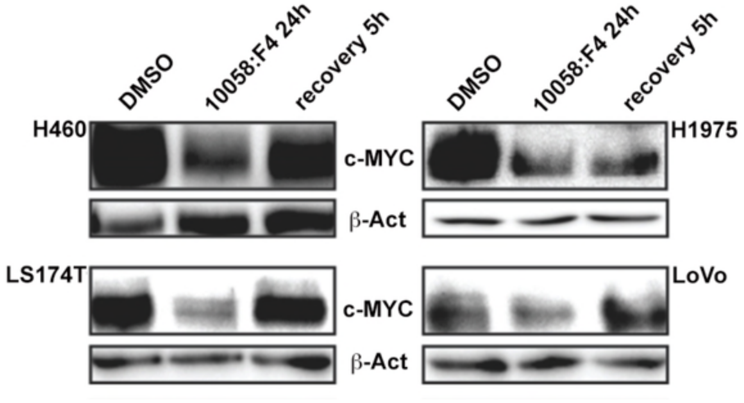

B

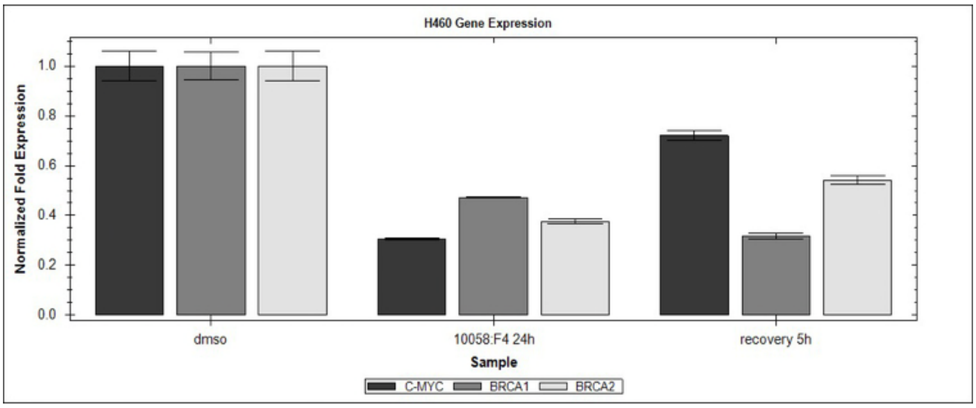

Supplementary Figure S11. A, Western Blotting showing c-MYC protein levels in human cell lines before treatment (DMSO), after 24h treatment with 60μM 10058:F4 (10058:F4 24h) and 5h after medium replacement (recovery 5h). β-actin is shown as a loading control. **B**, a representative qRT-PCR showing *c-MYC* and the MYC/MAX target genes *BRCA1* and *BRCA2* transcripts in H460 cell line before treatment (DMSO), after 24h treatment with 60μM 10058:F4 (10058:F4 24h) and 5h after medium replacement (recovery 5h). *GUSB* was used as reference gene. RNAs were extracted from samples homogenized in TRI-Reagent® (Sigma-Aldrich) with DNAse I treatment. cDNA synthesis was performed using the ThermoScript™ RT-PCR system (Invitrogen) and PCR was run in an iCycler Bio-Rad Real Time PCR Detection System using the SYBR GreenER qPCR SuperMix (Invitrogen).

Primers were as follow:

Gene	Primer sequences	Size (bp)	Melting (°C)	GenBank Accession
Homo sapiens				
c-MYC	Forward:<br>5'-GAGGAGGAACAAGAAGATGAGG-3'<br>Reverse:<br>5'-TCCAGCAGAAGGTGATCCA-3'	100	60	NM_002467.4
BRCA1	Forward:<br>5'-GGTGGTACATGCACAGTTGC-3'<br>Reverse:<br>5'-ACTCTGGGGCTCTGTCTTCA-3'	240	60	NM_007294.3
BRCA2	Forward:<br>5'-CCACAGCCAGGCAGTCTGTAT-3'<br>Reverse:<br>5'-AGAACACGCAGAGGGAACTTG-3'	96	60	NM_000059.3
GUSB	Forward:<br>5'-AGCGTGGAGCAAGACAGTGG-3'<br>Reverse:<br>5'-ATACAGATAGGCAGGGCGTTCG-3'	198	60	NM_000181.3

Di Giacomo et al. Supplementary Figure S12.

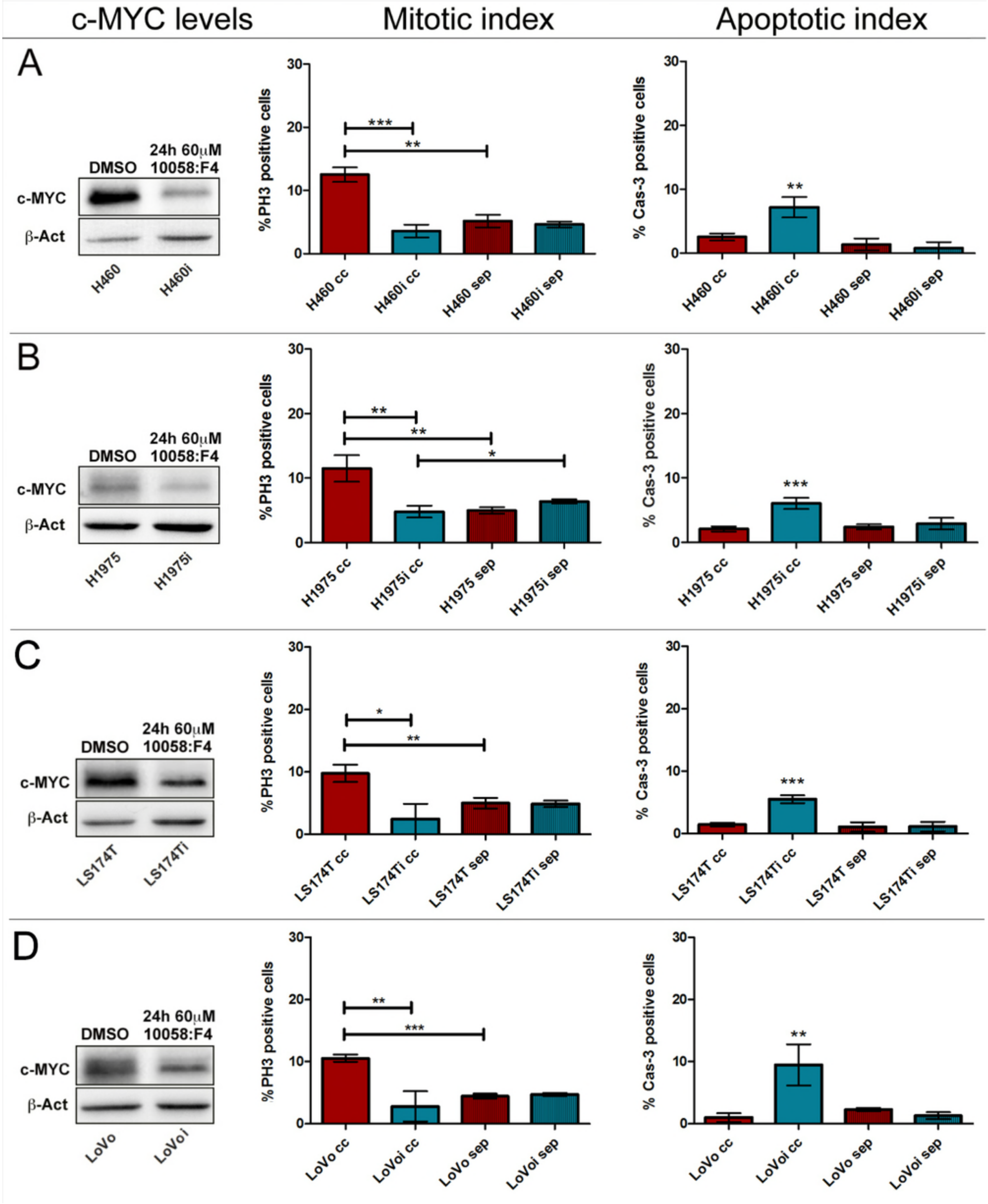

Supplementary Figure S12. Consequences of MYC level disparity in pairs of genetically identical cells. **A**, the inhibited H460 lung cancer line (H460i) shows a decrease in c-MYC protein levels and behaves as a loser in the competition assay. Consistently, it displays a higher percentage of Cas3-positive cells respect to the native line in co-culture (H460 cc) and to the two cell populations grown separately (H460 sep and H460i sep). Comparable results were obtained with the lung cancer cell line H1975 (**B**) and with the two colon cancer cell lines LS174T and LoVo (**C** and **D**). In each assay, the Western Blotting shows c-MYC protein levels in the untreated and treated cell lines and β-actin as a loading control, with the treatment indicated at the top of the lanes. The Mitotic Index shows the percentage of PH3-positive cells of the two lines after 5h in co-culture (cc) or in separate (sep) conditions; the Apoptotic Index is represented as the percentage of Cas3-positive cells at the end of the assay. Statistical significance and SD are indicated as an average from at least three independent replicates. *p*-values are: $p<0.05$ (*), $p<0.01$ (**) and $p<0.001$ (***).
